# Transfemoral Valve-in-Valve TAVI with MyVal for Failed Surgical Aortic Bioprostheses: Procedural Outcomes, Serial Hemodynamics, and Anatomy-Based Determinants of Residual Gradient

**DOI:** 10.3390/jcm15093462

**Published:** 2026-05-01

**Authors:** Georgios E. Papadopoulos, Ilias Ninios, Sotirios Evangelou, Andreas Ioannides, Athinodoros Nikitopoulos, Vlasis Ninios

**Affiliations:** Cardiology Department, Interbalkan Medical Center, 57001 Thessaloniki, Greece

**Keywords:** valve-in-valve TAVI, MyVal, ACURATE neo2, failed surgical bioprosthesis, transvalvular gradient, true internal diameter, hemodynamics, structural valve degeneration

## Abstract

**Background/Objectives:** Valve-in-valve transcatheter aortic valve implantation (ViV-TAVI) is an established treatment for failed surgical aortic bioprostheses, but dedicated data on the MyVal platform remain limited. We evaluated outcomes, hemodynamics, residual gradient, and an exploratory matched comparison with ACURATE neo2. **Methods:** This prospective, single-center cohort included consecutive patients with failed surgical aortic bioprostheses treated with MyVal ViV-TAVI between July 2022 and June 2025. Outcomes were reported according to VARC-3. **Results:** Sixty-eight patients were included (age 77 ± 7 years; 51.5% women; EuroSCORE II 7.3 ± 1.8%). Technical success was 100%, with no 30-day death, stroke, myocardial infarction, second-valve implantation, or emergency surgical conversion. Mean gradient decreased from 38.0 ± 9.5 mmHg at baseline to 6.7 ± 2.1 mmHg post-procedure and remained low at 1 year (8.1 ± 2.5 mmHg; overall *p* < 0.001). AVA increased from 0.80 ± 0.23 cm^2^ to 1.98 ± 0.19 cm^2^ post-procedure and was 1.86 ± 0.23 cm^2^ at 1 year (overall *p* < 0.001). Smaller true internal diameter independently predicted elevated 1-year gradient (adjusted OR per 1-mm decrease 1.33, 95% CI 1.04–1.78; *p* = 0.028). In the exploratory matched comparison, safety and hemodynamic outcomes did not differ significantly between MyVal and ACURATE neo2. At a median follow-up of 12.8 months, all-cause mortality and heart failure hospitalization were each 4.4%. **Conclusions:** In this prospective single-center cohort, MyVal ViV-TAVI was associated with high procedural success and favorable 1-year hemodynamic outcomes, with residual gradient driven mainly by small surgical valve true internal diameter.

## 1. Introduction

Bioprosthetic surgical aortic valve replacement is being used in an increasingly broad patient population, and structural deterioration of these prostheses is therefore becoming a more frequent clinical problem. For selected patients with failed surgical bioprosthetic aortic valves, valve-in-valve transcatheter aortic valve implantation (ViV-TAVI) has emerged as an established alternative to redo surgery and is now incorporated into contemporary guideline-based decision making [1,2,3,4].

Aortic ViV-TAVI remains a distinct procedural entity rather than a simple extension of native-valve TAVI because the failed surgical bioprosthesis may constrain transcatheter valve expansion and leave residual hemodynamic burden despite technically successful implantation [1,2,4]. This issue is especially relevant in small surgical bioprostheses, stenotic modes of structural valve degeneration, and in the presence of pre-existing prosthesis-patient mismatch, all of which have been associated with less favorable post-ViV physiology and outcomes [1,2,5,6,7,8]. Accordingly, contemporary ViV-TAVI requires meticulous preprocedural planning, with cardiac computed tomography playing a central role in defining surgical valve type, true internal diameter, coronary-risk anatomy, and the need for adjunctive strategies such as coronary protection or bioprosthetic valve fracture [3,9,10,11,12,13].

The MyVal transcatheter heart valve is a balloon-expandable platform with design characteristics that may be particularly relevant in the ViV setting, including an expanded sizing matrix with intermediate 1.5-mm size increments that may improve device-to-prosthesis matching in anatomically constrained procedures [14,15]. Although MyVal has shown encouraging performance in native aortic stenosis [16,17], MyVal-specific evidence in failed surgical bioprostheses remains limited. The earliest published experience consisted of a small case series, and subsequent multicenter data combined valve-in-valve and valve-in-ring procedures across left-sided valve positions rather than focusing specifically on serial hemodynamic performance after transfemoral aortic ViV-TAVI [18,19]. Accordingly, dedicated data on procedural outcomes, longitudinal valve performance, residual gradient burden, and the interaction between small-valve anatomy and optimization strategies in MyVal aortic ViV-TAVI remain sparse. On this background, the present study primarily evaluated consecutive patients undergoing transfemoral MyVal aortic ViV-TAVI at a high-volume structural heart center, with prespecified analyses focused on procedural safety, serial hemodynamic performance, and anatomy-based determinants of residual obstruction; an exploratory internal comparison with ACURATE neo2 was performed secondarily for institutional hemodynamic contextualization.

## 2. Materials and Methods

### 2.1. Study Design

This was a prospective, single-center, observational cohort study conducted at a high-volume structural heart disease center. The primary study cohort comprised consecutive adult patients with symptomatic structural degeneration of a previously implanted surgical bioprosthetic aortic valve who underwent transfemoral valve-in-valve (ViV) transcatheter aortic valve implantation (TAVI) with the MyVal transcatheter heart valve (Meril Life Sciences Pvt. Ltd., Vapi, India) between July 2022 and June 2025. The objectives of the present analysis were to evaluate procedural success, early safety, serial hemodynamic performance, and 1-year clinical outcomes after MyVal ViV-TAVI, with prespecified analyses focused on residual transvalvular gradients and anatomy-based subgroup effects; a secondary exploratory internal device-platform comparison was performed for contextualization.

All cases were evaluated by a multidisciplinary Heart Team that included interventional cardiologists, cardiac surgeons, cardiac imaging specialists, and cardiac anesthesiologists. Treatment decisions were based on clinical status, bioprosthetic failure mechanism, anatomical feasibility, procedural risk, and overall comorbidity burden in accordance with contemporary guideline-based practice. The study was conducted in accordance with the Declaration of Helsinki and was approved by the local institutional ethics committee. Written informed consent for the procedure and institutional use of clinical data was obtained according to local policy.

### 2.2. Patient Population

Eligible patients were adults with symptomatic dysfunction of a surgical bioprosthetic aortic valve who were treated with transfemoral ViV-TAVI using the MyVal platform during the study period. Structural valve degeneration was established using integrated clinical and imaging assessment and was classified according to the predominant failure mechanism as stenosis, regurgitation, or mixed dysfunction. The primary analysis was restricted to the consecutive MyVal-treated transfemoral ViV-TAVI cohort. Non-MyVal ViV procedures were not included in the primary cohort analysis, but an internal ACURATE neo2 (Boston Scientific, Marlborough, MA, USA) cohort was used separately for the exploratory matched comparative analysis.

Patients were not excluded from the primary MyVal cohort on the basis of small surgical valve size, coronary obstruction-risk anatomy, or anticipated need for adjunctive procedural strategies such as coronary protection, chimney stenting, or surgical valve fracture, because these features were intrinsic to the real-world anatomical complexity that this study sought to evaluate.

### 2.3. Preprocedural Assessment and Anatomical Planning

All patients underwent structured preprocedural clinical and imaging evaluation, including transthoracic echocardiography and invasive coronary angiography when clinically indicated. Contrast-enhanced electrocardiographically gated cardiac computed tomography was available in all 68 patients and was used systematically for ViV planning. Preprocedural imaging was used to characterize aortic root anatomy, identify the failed surgical bioprosthesis, assess coronary ostial height, sinus of Valsalva and sinotubular junction dimensions, evaluate valve-to-coronary relationships, and inform transcatheter valve sizing, implantation strategy, and the need for adjunctive measures such as coronary protection. Surgical valve true internal diameter (true ID) was determined from the known surgical prosthesis model and labeled size using manufacturer specifications and standard ViV planning references, with CT-based anatomical confirmation. For prespecified subgroup analyses, small surgical valves were defined as true ID ≤ 21 mm. Detailed case-level surgical valve characteristics are provided in Supplementary Table S1, and the distribution of key preprocedural CT planning parameters and coronary-risk features is summarized in Supplementary Table S2.

### 2.4. Procedure

All procedures were performed via transfemoral access under fluoroscopic guidance according to institutional practice. General anesthesia was used selectively rather than routinely and was reserved for procedurally more complex cases or when more controlled intraprocedural imaging, airway management, or hemodynamic support was considered preferable according to Heart Team, anesthesiology, and operator judgment. Valve sizing, implantation depth, and overall deployment strategy were based on preprocedural imaging, surgical valve identification, and anatomical risk assessment.

The procedural approach was anatomy-driven and allowed selective use of adjunctive techniques when indicated, including balloon predilation, postdilation, coronary protection, chimney stenting, and surgical valve fracture. These adjunctive strategies were not protocol-mandated and were used selectively at operator discretion to optimize valve expansion, minimize coronary risk, and reduce anticipated residual hemodynamic burden in anatomically constrained ViV procedures. In particular, attempted surgical valve fracture was considered only in selected cases after integrated review of the implanted surgical valve model and size, known or expected fracture amenability, small true internal diameter, anticipated constraint of transcatheter valve expansion, and overall procedural feasibility and safety. Because fracture selection was anatomy- and operator-dependent rather than randomized, any subsequent comparison between fracture and no-fracture subgroups was considered descriptive and non-causal. Procedural variables prospectively recorded included procedure duration, fluoroscopy time, contrast volume, use of adjunctive techniques, and intraprocedural complications. Because preservation of future coronary access formed part of the procedural planning framework, implantation orientation was optimized whenever feasible on the basis of anatomical considerations and device positioning strategy.

### 2.5. Follow-Up and Data Collection

Clinical and echocardiographic follow-up were prospectively scheduled at baseline, early post-procedure, 30 days, and 1 year. For each follow-up time point, the number of patients with available clinical and echocardiographic data was recorded. For patients without 1-year echocardiography, the reason for non-availability was classified as death before the 1-year visit, missed or unavailable echocardiographic assessment despite clinical follow-up, or complete loss to follow-up. Collected variables included baseline demographics, comorbidities, surgical valve characteristics, procedural details, in-hospital events, 30-day outcomes, survival status, heart failure hospitalization, repeat coronary angiography and/or percutaneous coronary intervention after ViV-TAVI, and New York Heart Association (NYHA) functional class at follow-up. Follow-up data were obtained from institutional records, outpatient assessments, and structured follow-up documentation.

### 2.6. Echocardiographic Assessment

Transthoracic echocardiography was performed according to routine institutional standards and interpreted by experienced echocardiographers using standard multiparametric prosthetic valve assessment principles. Formal interobserver reproducibility analysis and core-laboratory adjudication were not prospectively incorporated into the study design. For the present analysis, the prespecified core hemodynamic variables were mean transvalvular gradient and aortic valve area (AVA).

Mean transvalvular gradient was derived from continuous-wave Doppler interrogation of the aortic prosthesis using standard Bernoulli-based methods. AVA was calculated by the continuity equation. Measurements were recorded at baseline, post-procedure, 30 days, and 1 year. At 1 year, valve competence was additionally described by grading total aortic regurgitation and paravalvular leak (PVL), and the proportions with greater-than-mild regurgitation or PVL were reported descriptively. For echocardiographic follow-up endpoints, percentages were calculated using the number of patients with available echocardiography at the relevant time point. This available-case approach also applied to subgroup analyses of 1-year echocardiographic outcomes.

### 2.7. Outcomes and Endpoint Definitions

Procedural and early clinical outcomes were assessed using Valve Academic Research Consortium-3 (VARC-3) definitions where applicable. Technical success was reported according to VARC-3 criteria [20]. Early safety at 30 days was summarized using a VARC-3-based composite, and the individual 30-day adverse events reported in the present manuscript included all-cause mortality, stroke, myocardial infarction, major vascular complications, major or life-threatening bleeding, acute kidney injury stage 2–3, new permanent pacemaker implantation, valve dysfunction requiring repeat procedure, and valve-related reintervention.

The primary hemodynamic effectiveness analyses focused on serial changes in mean transvalvular gradient and AVA from baseline to post-procedure, 30 days, and 1 year. In addition, a prespecified binary residual hemodynamic endpoint for exploratory risk-factor analysis was mean transvalvular gradient ≥ 10 mmHg at 1-year echocardiography. This threshold was selected as a conservative and clinically interpretable marker of any nontrivial residual hemodynamic burden. We emphasize that this cutoff was not intended to represent a formal consensus definition of high residual gradient after ViV-TAVI; rather, it was chosen in light of prior ViV literature showing that clearly elevated residual gradients are more commonly defined at ≥20 mmHg, while recent analyses have examined outcomes across gradient strata including <10, 10–20, 20–30, and ≥30 mmHg [8,20]. In addition, standardized definitions of bioprosthetic hemodynamic deterioration use a 10 mmHg increase in mean gradient as a clinically meaningful marker of worsening valve hemodynamics. Because dichotomization reduces information, particularly in a modest-sized cohort, continuous gradient analyses were considered primary, and the binary threshold-based endpoint was treated as complementary.

Clinical follow-up outcomes included all-cause mortality, heart failure hospitalization, endocarditis, valve thrombosis, valve-related reintervention, and NYHA functional class. Post-ViV coronary access was assessed pragmatically only in cases in which selective coronary angiography and/or PCI was attempted during follow-up; these observations were considered opportunistic and were not interpreted as proof of preserved future coronary access across the full cohort.

### 2.8. Prespecified Subgroup Analyses

Two prespecified subgroup analyses were performed to evaluate anatomy- and strategy-related determinants of residual hemodynamic burden. First, the primary MyVal cohort was stratified according to surgical valve true ID (≤21 mm vs. >21 mm) to examine differences in serial gradients, 1-year echocardiographic valve performance, and clinical outcomes. Second, within the small-valve subgroup (true ID ≤ 21 mm), outcomes were described according to whether surgical valve fracture was attempted, with the aim of exploring the hemodynamic profile of fracture-selected versus non-fracture-treated anatomically constrained cases. Because fracture was not randomly assigned and was strongly influenced by surgical valve characteristics, anatomical suitability, and operator judgment, this analysis was not intended to estimate a causal treatment effect. Given the limited subgroup sample sizes and event counts, these analyses were considered descriptive, mechanistic, and hypothesis-generating. Emphasis was therefore placed on effect size, directionality, and internal consistency across related hemodynamic endpoints rather than on isolated nominal *p*-values alone.

### 2.9. Exploratory Internal Device-Platform Comparison

To contextualize MyVal performance within local ViV-TAVI practice, an exploratory internal comparative analysis was performed against a non-randomized institutional cohort of transfemoral ViV-TAVI procedures treated with ACURATE neo2. ACURATE neo2 was selected as the exploratory comparator because it represented the principal supra-annular self-expanding ViV-TAVI platform used contemporaneously at our institution during the study era. Because device selection was influenced by anatomy and treatment era, crude comparisons were considered susceptible to confounding by indication. Propensity score methods were therefore used to improve comparability between device groups within the region of covariate overlap. Propensity scores for treatment with MyVal were estimated using multivariable logistic regression including prespecified baseline clinical variables (age, sex, body mass index, EuroSCORE II, renal function/chronic kidney disease category, left ventricular ejection fraction, atrial fibrillation, coronary artery disease, prior PCI, prior CABG, prior stroke/TIA, chronic obstructive pulmonary disease, and baseline NYHA class), valve and hemodynamic variables (predominant failure mechanism, baseline mean transvalvular gradient, baseline aortic valve area, and surgical valve true internal diameter), anatomical/coronary-risk phenotype, and treatment era. The primary comparative analysis used 1:1 nearest-neighbor matching without replacement, with restriction to the region of common support. Covariate balance was assessed using standardized mean differences before and after matching, and overlap and balance diagnostics are now shown in the Supplementary Material, including a pre- versus post-match balance table, a Love plot, and a propensity score overlap figure. Complementary sensitivity analyses, including overlap weighting and residual covariate-adjusted analyses when indicated, are also summarized in the Supplementary Material.

### 2.10. Statistical Analysis

All analyses were performed using R (R Foundation for Statistical Computing, Vienna, Austria) (version 2025.09.2+418). Continuous variables are reported as mean ± standard deviation or median with interquartile range, as appropriate, and categorical variables as counts and percentages. All statistical tests were two-sided, and a *p*-value < 0.05 was considered statistically significant. Because subgroup and device-platform comparisons were exploratory, *p*-values from these analyses were interpreted descriptively and were not adjusted for multiple comparisons.

For between-group comparisons in the primary cohort and prespecified subgroup analyses, continuous variables were compared using Student’s *t*-test or Welch’s *t*-test when assumptions for parametric testing were judged acceptable and by Mann-Whitney U testing otherwise. Categorical variables were compared using the chi-square test or Fisher’s exact test, as appropriate according to cell counts.

Serial hemodynamic outcomes, including mean transvalvular gradient and AVA at baseline, post-procedure, 30 days, and 1 year, were analyzed using linear mixed-effects models with a patient-level random intercept to account for within-patient correlation across repeated measurements. Time was modeled as a categorical fixed effect. Overall time effects and prespecified pairwise comparisons between post-procedure, 30-day, and 1-year time points were derived from model-based marginal means.

Predictors of residual hemodynamic burden were evaluated using logistic regression for the exploratory binary endpoint of mean transvalvular gradient ≥ 10 mmHg at 1 year among patients with available 1-year echocardiography. Because of the limited number of events, Firth penalized logistic regression was used to reduce small-sample bias and instability related to sparse data or quasi-separation. Univariable models were followed by a prespecified multivariable model including surgical valve true ID as a continuous variable, baseline mean transvalvular gradient, failure mechanism category, and surgical valve fracture strategy. Odds ratios (ORs) with 95% confidence intervals (CIs) are reported, and the effect of true ID is expressed per 1-mm decrease. An exploratory true ID × fracture interaction was examined in a secondary model.

For the exploratory MyVal versus ACURATE neo2 comparison, propensity scores for treatment with MyVal were estimated using multivariable logistic regression including clinically relevant baseline, anatomical, and treatment-era variables associated with device selection and outcomes. One-to-one nearest-neighbor matching without replacement was performed on the logit of the propensity score using a caliper width of 0.2 standard deviations of the logit of the propensity score and restriction to the region of common support. Covariate balance after matching was assessed using standardized mean differences, with an absolute standardized mean difference <0.10 considered acceptable.

In the matched cohort, continuous fixed-time outcomes were analyzed using matched-pair methods and are reported as mean differences with 95% CIs. Binary fixed-time outcomes were analyzed using matched-data methods appropriate for paired samples, with effect estimates reported as ORs when estimable. Longitudinal matched hemodynamic trajectories were additionally examined using mixed-effects models including fixed effects for time, device platform, and the time-by-device interaction. Time-to-event outcomes during follow-up, including all-cause mortality, heart failure hospitalization, and the composite of all-cause mortality or heart failure hospitalization, were analyzed exploratorily using Cox proportional hazards models stratified by matched pair, with hazard ratios and 95% CIs reported. Given the low number of clinical follow-up events, these time-to-event estimates were considered exploratory and interpreted descriptively.

### 2.11. Missing Data

Missing data were handled using an analysis-specific available-data approach. Serial hemodynamic analyses were performed using linear mixed-effects models estimated with maximum likelihood, thereby incorporating all available repeated measurements without requiring complete follow-up at every time point under a missing-at-random assumption. Cross-sectional follow-up analyses, including the 1-year residual gradient endpoint and subgroup-specific 1-year echocardiographic comparisons, were conducted as available-case analyses using the corresponding time-point denominators. No imputation was performed.

## 3. Results

### 3.1. Baseline Characteristics

**Figure 1:**
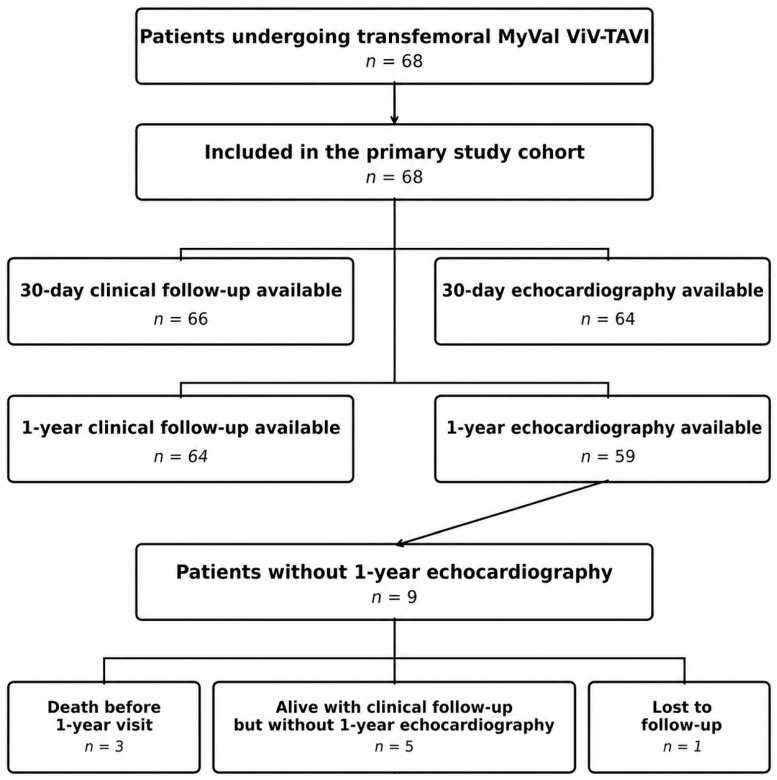
Flow of patients and availability of clinical and echocardiographic follow-up in the MyVal ViV-TAVI cohort.

A total of 68 patients (51.5% female) underwent transfemoral ViV-TAVI with MyVal. Mean age at baseline was 77 ± 7 years (Table 1). The cohort was at high surgical risk, with a mean EuroSCORE II of 7.3 ± 1.8%, and had a substantial comorbidity burden; hypertension (77.9%) and dyslipidemia (66.2%) were the most prevalent baseline conditions. At presentation, 26/68 patients (38.0%) were in NYHA class IV. Stenosis was the predominant mechanism of surgical bioprosthetic failure, accounting for 34/68 cases (50.0%). Mean surgical valve true internal diameter (true ID) was 22 ± 3 mm, and 24/68 patients (35.3%) had small surgical valves (true ID ≤ 21 mm). At the time of analysis, 59 of 68 patients had available 1-year echocardiography. Among the 9 patients without 1-year echocardiographic data, 3 had died before the 1-year visit, 5 were alive with clinical follow-up but without an available echocardiographic examination, and 1 was lost to follow-up (Figure 1). Accordingly, all 1-year echocardiographic analyses, including the residual-gradient endpoint and subgroup comparisons, were performed using available-case denominators.

### 3.2. Procedural Characteristics

All procedures were performed through transfemoral access (100%) (Table 2). General anesthesia was used in 19/68 procedures (27.9%) and reflected selective use in more complex cases rather than a routine procedural strategy. Predilation and postdilation were used in 42.6% and 26.5% of cases, respectively, whereas surgical valve fracture was performed in 23.5%, predominantly in small-valve anatomies. Coronary protection and chimney stenting were used selectively in 11.8% and 5.9% of procedures, respectively. No patient required second-valve implantation or emergency surgical conversion, and no unresolved intraprocedural coronary flow compromise occurred.

### 3.3. Procedural Success and 30-Day Outcomes

Procedural success was high, with VARC-3 technical success achieved in all 68/68 patients (Table 3). The VARC-3-based early safety composite at 30 days was 4/68 (5.9%). Individual 30-day adverse events were infrequent: there were no deaths, strokes, or myocardial infarctions, while major vascular complications, major/life-threatening bleeding, and AKI stage 2–3 each occurred in 1/68 patients (1.5%), and new permanent pacemaker implantation occurred in 2/68 patients (2.9%).

### 3.4. Serial Hemodynamic Performance

**Figure 2:**
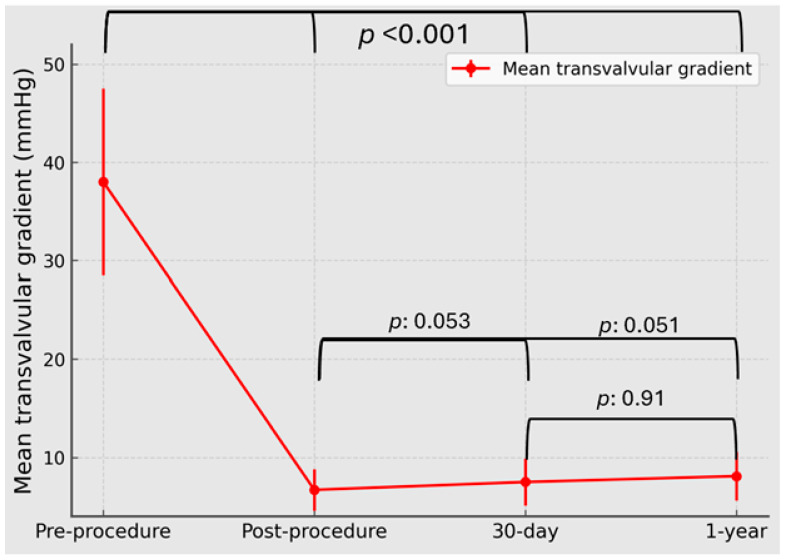
Serial mean transvalvular gradient after MyVal ViV-TAVI. Mean transvalvular gradient at baseline, post-procedure, 30 days, and 1 year after transfemoral MyVal valve-in-valve transcatheter aortic valve implantation. Values are shown as mean ± SD. Overall time effect: *p* < 0.001. Pairwise comparisons: post-procedure vs. 30 days, *p* = 0.053; post-procedure vs. 1 year, *p* = 0.051; 30 days vs. 1 year, *p* = 0.91. Observed echocardiographic sample sizes contributing data at each time point were n = 68 at baseline, n = 68 post-procedure, n = 64 at 30 days, and n = 59 at 1 year.

**Figure 3:**
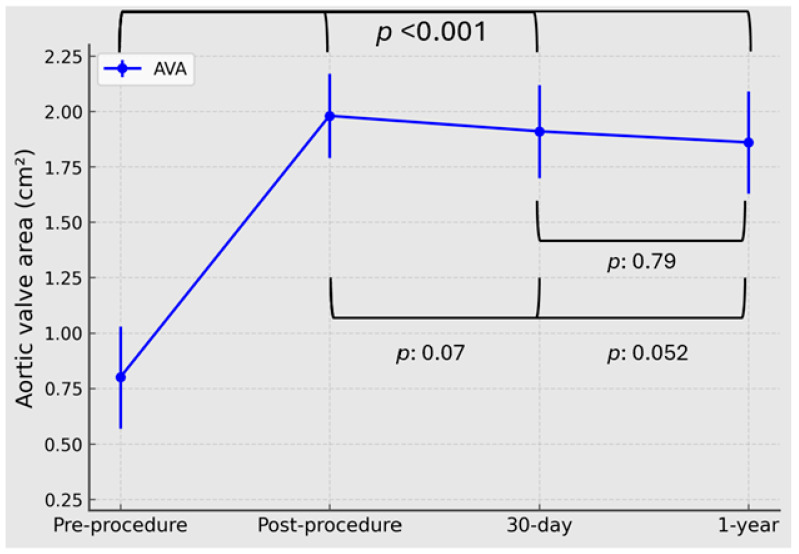
Serial aortic valve area after MyVal ViV-TAVI. Aortic valve area at baseline, post-procedure, 30 days, and 1 year after transfemoral MyVal valve-in-valve transcatheter aortic valve implantation. Values are shown as mean ± SD. Overall time effect: *p* < 0.001. Pairwise comparisons: post-procedure vs. 30 days, *p* = 0.07; post-procedure vs. 1 year, *p* = 0.052; 30 days vs. 1 year, *p* = 0.79. Observed echocardiographic sample sizes contributing data at each time point were n = 68 at baseline, n = 68 post-procedure, n = 64 at 30 days, and n = 59 at 1 year.

Serial echocardiography showed marked immediate hemodynamic improvement after MyVal ViV-TAVI, with preservation of valve performance through 1 year. Observed echocardiographic sample sizes were n = 68 at baseline, n = 68 post-procedure, n = 64 at 30 days, and n = 59 at 1 year. Mean transvalvular gradient decreased from 38.0 ± 9.5 mmHg at baseline to 6.7 ± 2.1 mmHg post-procedure and remained low at follow-up, measuring 7.5 ± 2.4 mmHg at 30 days and 8.1 ± 2.5 mmHg at 1 year (overall *p* < 0.001) (Figure 2). The small numerical increases observed after the post-procedural study did not translate into significant pairwise differences between post-procedure and 30 days (*p* = 0.053), post-procedure and 1 year (*p* = 0.051), or 30 days and 1 year (*p* = 0.91). In parallel, AVA increased from 0.80 ± 0.23 cm^2^ at baseline to 1.98 ± 0.19 cm^2^ post-procedure and remained preserved at 30 days (1.91 ± 0.21 cm^2^) and 1 year (1.86 ± 0.23 cm^2^; overall *p* < 0.001; post-procedure vs. 30 days *p* = 0.07, post-procedure vs. 1 year *p* = 0.052, and 30 days vs. 1 year *p* = 0.79) (Figure 3).

**Figure 4:**
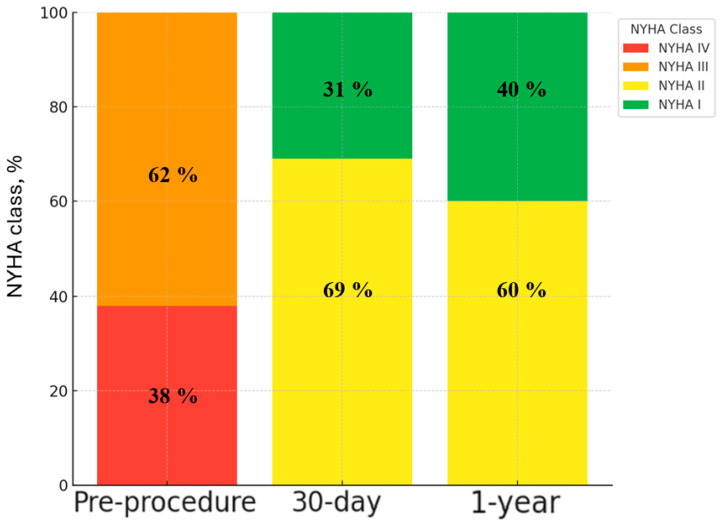
Distribution of NYHA functional class at baseline and follow-up after MyVal ViV-TAVI. Stacked percentage distribution of NYHA functional class at baseline, 30 days, and 1 year after transfemoral MyVal valve-in-valve transcatheter aortic valve implantation, showing a shift from baseline NYHA III-IV to follow-up NYHA I-II. Percentages are shown using the number of patients with available NYHA data at each time point as the denominator: baseline n = 68, 30-day n = 66, and 1-year n = 64.

Among patients with available 1-year echocardiography (*n* = 59), a mean gradient ≥ 10 mmHg was observed in 23.7%. Significant regurgitation remained uncommon, with greater-than-mild total aortic regurgitation in 2/59 patients (3.4%) and greater-than-mild PVL in 1/59 (1.7%). At a median follow-up of 12.8 months, all-cause mortality and heart failure hospitalization were each 4.4%, and no cases of endocarditis, valve thrombosis, or valve-related reintervention were recorded. Functional status improved substantially over follow-up. A patient-level summary of all follow-up adverse events, including event timing and valve-status context, is provided in Supplementary Table S5. Whereas all patients were in NYHA class III-IV at baseline, among patients with available NYHA assessment at 30 days (*n* = 66), 46/66 (69.7%) were in class II and 20/66 (30.3%) in class I. Among patients with available NYHA assessment at 1 year (*n* = 64), 38/64 (59.4%) were in class II and 26/64 (40.6%) in class I (Figure 4).

### 3.5. Subgroup Analysis According to Surgical Valve True Internal Diameter

**Figure 5:**
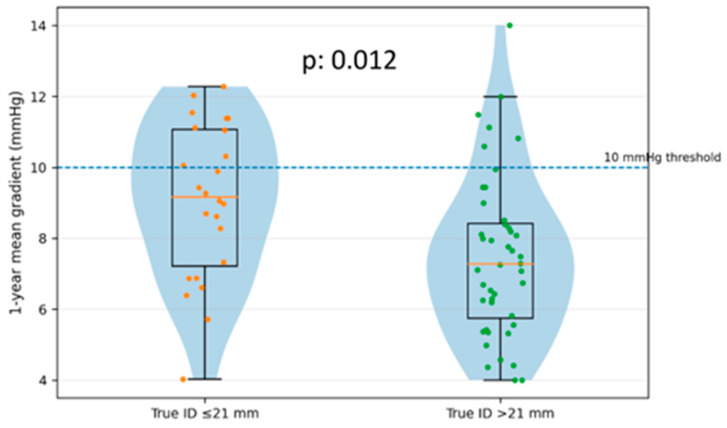
Distribution of 1-year mean transvalvular gradient according to surgical valve true internal diameter. Violin plot with overlaid boxplot and individual observations showing the distribution of 1-year mean transvalvular gradient in patients with surgical valve true ID ≤ 21 mm versus >21 mm after MyVal ViV-TAVI. The dashed line indicates the 10 mmHg threshold for elevated residual gradient. Patients with true ID ≤ 21 mm had higher 1-year mean gradients than those with true ID > 21 mm (9.1 ± 2.6 vs. 7.6 ± 2.2 mmHg; *p* = 0.012). The violin (blue area) shows the data density, the boxplot shows median (orange line) and IQR, and dots (orange and green) represent individual patients.

Surgical valve true internal diameter (true ID) was significantly associated with residual hemodynamic burden (Table 4). Patients with small surgical valves (true ID ≤ 21 mm) had higher 1-year mean transvalvular gradients than those with larger valves (9.1 ± 2.6 vs. 7.6 ± 2.2 mmHg, *p* = 0.012) and more frequently exhibited a mean gradient ≥ 10 mmHg at 1 year (9/22 [40.9%] vs. 5/37 [13.5%], *p* = 0.026). Figure 5 illustrates that the true ID ≤ 21 mm subgroup had a distribution of 1-year mean gradients centered at higher values, with a greater proportion of observations above the 10 mmHg threshold. Surgical valve fracture was performed more frequently in small-valve anatomies (58.3% vs. 4.5%, *p* < 0.001), consistent with the anatomy-driven procedural strategy. Despite the higher residual gradient burden in patients with small surgical valves, 30-day early safety and follow-up clinical event rates did not differ significantly between the true ID subgroups.

### 3.6. Small-Valve Subgroup: Descriptive Analysis According to Fracture Strategy

Within the small-valve subgroup (true ID ≤ 21 mm), patients selected for surgical valve fracture showed a more favorable observed hemodynamic profile than those managed without fracture (Table 5, Supplementary Figure S3). Among the 14 small-valve patients undergoing bioprosthetic valve fracture, the underlying surgical bioprosthesis models were Magna Ease (*n* = 4), Perimount 2800 (*n* = 3), Mosaic (*n* = 2), Mitroflow (*n* = 2), Biocor Epic (*n* = 2), and Magna (*n* = 1); detailed model-specific and size-specific data are provided in Supplementary Table S1. Patients undergoing fracture had lower mean gradients immediately after the procedure (6.1 ± 1.7 vs. 7.6 ± 2.0 mmHg, *p* = 0.041) and at 1 year (8.0 ± 2.1 vs. 10.4 ± 2.7 mmHg, *p* = 0.028). The 30-day mean gradient also tended to be lower in the fracture group (7.2 ± 2.0 vs. 9.1 ± 2.6 mmHg, *p* = 0.056). The proportion of patients with mean gradient ≥ 10 mmHg at 1 year was numerically lower after fracture, although this difference did not reach conventional statistical significance (3/13 [23.1%] vs. 6/9 [66.7%], *p* = 0.079). Clinical event rates remained low in both groups.

### 3.7. Predictors of Elevated Residual Gradient at 1 Year

The anatomy-centered signal observed in the continuous gradient analyses was reinforced in exploratory multivariable modeling of the binary residual-gradient endpoint (Table 6). In Firth penalized logistic regression for elevated 1-year gradient (≥10 mmHg), smaller true ID remained the only independent predictor after adjustment for baseline gradient, failure mechanism, and fracture strategy (adjusted OR per 1-mm decrease 1.33, 95% CI 1.04–1.78, *p* = 0.028). Baseline mean gradient was not independently associated with the outcome, and fracture showed a directionally protective but non-significant association. In exploratory testing, the true ID × fracture interaction was not significant (pinteraction = 0.12).

### 3.8. Exploratory Matched Comparison with ACURATE neo2

**Figure 6:**
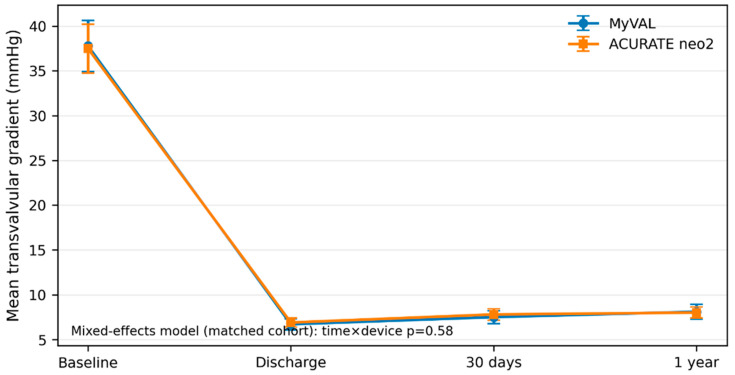
Serial mean transvalvular gradient in the matched MyVal and ACURATE neo2 cohorts. Mean transvalvular gradient at baseline, discharge, 30 days, and 1 year in the propensity score-matched MyVal and ACURATE neo2 cohorts after valve-in-valve TAVI. Values are shown as mean ± SD. The time × device *p* value derives from the mixed-effects model for longitudinal gradient trajectories.

To contextualize MyVal performance within local ViV practice, exploratory matched analyses were performed against an internal ACURATE neo2 cohort (Table 7, Figure 6). For the exploratory comparative analysis, the initial cohort comprised 68 MyVal-treated and 49 ACURATE neo2-treated transfemoral ViV-TAVI procedures. After assessment of propensity-score overlap, 6 MyVal cases and 3 ACURATE neo2 cases were excluded outside the region of common support, leaving 62 and 46 patients eligible for matching, respectively. One-to-one nearest-neighbor propensity-score matching without replacement then yielded 38 matched pairs. Measured covariate balance improved substantially after matching, with all post-match absolute standardized mean differences <0.10. Pre- and post-match balance summaries are provided in Supplementary Table S3 and Supplementary Figure S1, and propensity-score overlap/common-support diagnostics are shown in Supplementary Figure S2. In the matched cohort of 38 pairs, procedural success and 30-day safety remained high in both groups. No 30-day deaths, strokes, or myocardial infarctions occurred in either platform group. There was no clear between-platform difference in fixed-time hemodynamic outcomes, with post-procedure mean gradients of 6.7 ± 2.0 versus 6.9 ± 1.6 mmHg (*p* = 0.62), 30-day mean gradients of 7.5 ± 2.2 versus 7.8 ± 1.9 mmHg (*p* = 0.48), and 1-year mean gradients of 8.1 ± 2.4 versus 8.0 ± 1.8 mmHg (*p* = 0.84) for MyVal and ACURATE neo2, respectively. The proportion with mean gradient ≥ 10 mmHg at 1 year was likewise similar (21.1% vs. 23.7%, *p* = 0.79). Longitudinal trajectories remained closely overlapping, and mixed-effects modeling showed no evidence of differential gradient evolution over time between platforms. Follow-up clinical comparisons were limited by low event counts and wide confidence intervals, but no statistically significant between-platform differences were observed. In attempted follow-up cases only, post-ViV coronary access was feasible in all 14 MyVal cases and in 10 of 11 ACURATE neo2 cases (*p* = 0.26). In sensitivity analyses, overlap-weighted, doubly robust adjusted, and restricted-era models yielded estimates that were directionally concordant with the primary matched analysis and did not materially alter the overall interpretation of the comparative findings (Supplementary Table S4).

## 4. Discussion

The principal findings of the present study are fourfold. First, in a prospective, consecutive real-world cohort, MyVal valve-in-valve transcatheter aortic valve implantation (ViV-TAVI) was associated with excellent procedural success and a very low burden of early adverse events despite substantial anatomic complexity, including a meaningful proportion of small surgical bioprostheses and selective use of coronary protection, chimney stenting, and surgical valve fracture. Second, MyVal implantation was associated with immediate hemodynamic improvement, with a marked fall in mean transvalvular gradient, generally preserved aortic valve area through 1 year among patients with available echocardiographic follow-up, and parallel improvement in functional status. Third, residual hemodynamic burden was driven primarily by the structural constraint imposed by the failed surgical bioprosthesis, with true internal diameter (true ID) emerging as the dominant determinant of 1-year residual gradient, whereas baseline gradient severity was not independently predictive after adjustment. Fourth, within the limits of a secondary exploratory matched comparison used for institutional contextualization, no statistically significant between-platform differences were observed in early safety, fixed-time 1-year hemodynamic measures, or longitudinal gradient trajectories. These findings are descriptive and should not be interpreted as establishing equivalence or formal comparability between MyVal and ACURATE neo2. Taken together, these findings provide preliminary single-center evidence that MyVal can be used feasibly in contemporary ViV-TAVI practice, while reinforcing the broader principle that post-ViV physiology is determined largely by anatomy rather than by device label alone [1,2,4,18,19].

From a safety standpoint, the present data compare favorably with the major historical ViV-TAVI experiences. In the Global Valve-in-Valve Registry, procedural success was 93.1% and 30-day mortality was 8.4%, with device malposition, coronary obstruction, and residual gradients identified as the central limitations of early ViV practice [1]. The STS/ACC Registry subsequently confirmed the effectiveness of ViV-TAVI at scale, and the PARTNER 2 aortic ViV registry showed sustained clinical and echocardiographic benefit at 5 years in high-risk patients [2,4]. In that context, the present study’s absence of 30-day death, stroke, or myocardial infarction and the absence of second-valve implantation or emergency conversion is consistent with the progressive maturation of ViV practice through better computed tomography planning, improved transcatheter heart valve technology, more selective use of adjunctive techniques, and greater operator familiarity with bioprosthetic failure phenotypes [1,2,4].

The hemodynamic data are particularly important because residual obstruction remains the Achilles heel of ViV-TAVI. In the present cohort, mean gradient fell from 38.0 mmHg at baseline to 6.7 mmHg post-procedure and remained low at 30 days and 1 year, while aortic valve area nearly doubled immediately after implantation and was preserved over follow-up. These values compare favorably with early ViV experience, including the Global Valve-in-Valve Registry, in which postprocedural mean gradients were substantially higher overall, and they also extend the very limited MyVal-specific ViV literature, which previously consisted mainly of an initial case series and a later multicenter left-sided ViV/valve-in-ring report that was not designed as a dedicated transfemoral aortic hemodynamic study [1,18,19]. The present results therefore add granularity that was previously missing from the MyVal literature: not only was implantation feasible, but the achieved valve performance was sustained through serial follow-up rather than being confined to the index procedure alone [1,18,19].

Small surgical bioprostheses (true ID ≤ 21 mm) exhibited higher 1-year continuous gradients and more frequently exceeded the prespecified ≥10 mmHg threshold used as a conservative marker of residual hemodynamic burden. We do not intend this cutoff to represent a formal consensus definition of high residual gradient after ViV-TAVI; rather, it was used as a lower-bound indicator of nontrivial residual obstruction, below the more established ≥20 mmHg threshold commonly applied in ViV studies and device-success frameworks [8]. These findings are consistent with prior ViV literature. Dvir et al. showed that smaller surgical valves and stenotic failure modes are associated with less favorable post-ViV hemodynamics and survival [6], whereas Pibarot et al. demonstrated that severe pre-existing prosthesis–patient mismatch of the failed surgical valve is independently associated with excess mortality after ViV implantation and with a higher likelihood of elevated postprocedural gradients [7]. Taken together, these observations support the concept that post-ViV physiology is determined primarily by the fixed structural constraints of the failed surgical bioprosthesis. In this framework, true internal diameter should not be viewed as a simple geometric descriptor, but as an integrative marker of residual hemodynamic risk, the functional consequences of pre-existing prosthesis–patient mismatch, and the feasibility of optimization strategies in anatomically constrained cases. The absence of an independent association between baseline gradient severity and 1-year residual gradient in our adjusted analysis further reinforces the view that final hemodynamic performance after ViV implantation is governed predominantly by host bioprosthetic anatomy rather than by the baseline Doppler gradient itself [6,7]. These considerations also have practical implications for valve selection and procedural planning: in small true-ID anatomies, careful device/prosthesis matching, CT-based assessment, and selective use of adjunctive strategies may be required to mitigate persistent residual obstruction. Although any single threshold is necessarily pragmatic, the concordance between the continuous and threshold-based analyses strengthens the interpretation that residual hemodynamic burden after ViV-TAVI is fundamentally anatomy driven.

The fracture analysis should be interpreted in the same anatomy-first framework and requires particularly cautious interpretation. Although descriptively lower post-procedural and 1-year gradients were observed in the fracture-selected subgroup of patients with small surgical bioprostheses, this comparison is highly vulnerable to selection effects and should not be interpreted causally. Fracture was neither randomized nor protocol-mandated, but rather was used selectively in anatomically chosen cases according to surgical valve model, expected fracture amenability, anticipated hemodynamic constraint, and operator judgment. Accordingly, the observed differences may reflect a combination of case selection, surgical valve type, concomitant optimization strategies such as postdilation, and other measured or unmeasured procedural factors rather than the fracture maneuver itself. This direction of effect is concordant with the original bioprosthetic valve fracture literature, which showed that fracturing the surgical valve ring can expand the effective orifice and improve post-ViV hemodynamics [13]. However, contemporary registry data have appropriately tempered indiscriminate enthusiasm. In the TVT Registry analysis limited to balloon-expandable SAPIEN 3/Ultra ViV-TAVR, attempted bioprosthetic valve fracture was associated with higher in-hospital mortality and life-threatening bleeding, while the hemodynamic gains, although real, were modest overall [12]. Within small surgical bioprostheses, fracture was associated with lower residual gradients, consistent with a potential role in mitigating anatomically constrained post-valve-in-valve hemodynamics. These findings should not be interpreted as supporting routine fracture, but rather as suggesting benefit in selected high-risk anatomies. Given the limited subgroup size and the absence of a significant interaction, the results remain exploratory and should be viewed as hypothesis-generating rather than definitive [12,13].

The exploratory matched comparison with ACURATE neo2 offers additional perspective on the hemodynamic profile of MyVal in valve-in-valve TAVI. This comparison should be understood as a historical internal benchmark against a contemporaneously used supra-annular self-expanding platform rather than as an attempt to guide current device selection, particularly because ACURATE neo2 is no longer commercially available. A supra-annular self-expanding platform would theoretically be expected to provide lower residual gradients than a balloon-expandable device, particularly in small failed surgical bioprostheses. Prior comparative studies and the LYTEN program support this expectation, having shown lower postprocedural and 1-year gradients with self-expanding valves, although without clear differences in short- to intermediate-term clinical outcomes [21,22,23,24]. Against this background, the absence of statistically significant hemodynamic differences between MyVal and ACURATE neo2 in the matched cohort is noteworthy but should be interpreted cautiously. This may reflect more granular prosthesis matching enabled by the intermediate MyVal sizes, anatomy-driven use of adjunctive optimization strategies, and the limited ability of a modest matched analysis to fully account for confounding by indication in a procedure where device choice is closely linked to surgical valve type, coronary anatomy, and operator preference [16,17,21,22,23]. These findings therefore support the feasibility of achieving comparable hemodynamic results with MyVal in selected anatomies but should not be interpreted as establishing equivalence between balloon-expandable and supra-annular self-expanding platforms in valve-in-valve TAVI [16,17,21,22,23].

The favorable coronary results in the present cohort occurred within a contemporary CT-guided, anatomy-based ViV-TAVI strategy. Because coronary obstruction and loss of future coronary access remain major limitations of valve-in-valve intervention, detailed preprocedural assessment of valve-to-coronary relationships, root anatomy, and surgical prosthesis characteristics is essential for procedural planning and for selection of preventive measures [9,10]. Within this framework, the absence of unresolved intraprocedural coronary compromise and the high feasibility of coronary access in attempted cases are most likely attributable to systematic risk stratification and selective use of adjunctive techniques, including coronary protection, chimney stenting, and alignment-focused implantation [9,10,11,25]. The favorable coronary-access observations in the present study were limited to cases in which coronary access was actually attempted during follow-up and should therefore be interpreted cautiously as pragmatic observations rather than definitive evidence that future coronary access was preserved.

An additional perspective not directly addressed by the present study is the potential role of exercise-based functional hemodynamic assessment. In aortic stenosis, exercise stress echocardiography can unmask latent symptoms and abnormal exercise-related hemodynamic responses not evident at rest and may contribute to risk stratification and timing of intervention [26]. In the broader TAVI population, emerging data also suggest that exercise-related functional and hemodynamic indices may provide incremental information beyond resting assessment [27]. This concept may be particularly relevant after valve-in-valve procedures, where residual obstruction is often anatomy constrained and resting gradients may not fully capture exertional physiology or functional limitation. Although exercise stress echocardiography was not part of the present study protocol, this represents an important area for future investigation and may help refine follow-up and clinical decision-making after valve-in-valve TAVI.

Several limitations should be considered when interpreting these findings. First, this was a prospective, single-center observational study without external event adjudication, formal interobserver reproducibility analysis, or core-laboratory echocardiographic assessment, which may limit imaging standardization and generalizability. Second, although the cohort was assembled consecutively and prospectively, the overall sample size was modest, particularly for subgroup, multivariable, and exploratory comparative analyses, thereby limiting statistical precision. Accordingly, the small-valve subgroup analysis and, in particular, the fracture versus no-fracture analysis should be regarded as strictly descriptive and hypothesis-generating. Attempted fracture was not randomly assigned or protocol-mandated, but was selected according to surgical valve type, anatomical suitability, anticipated hemodynamic constraint, procedural feasibility, and operator judgment; therefore, the more favorable hemodynamic findings observed in fracture-selected cases cannot be interpreted as causal and may reflect confounding by indication, valve-specific characteristics, concomitant optimization strategies, or other measured and unmeasured procedural factors. Third, 1-year echocardiographic outcomes were analyzed using an available-case approach rather than complete follow-up in all enrolled patients. Because the principal residual-gradient endpoint and related subgroup analyses were based on the 1-year echocardiographic subset, these findings should be interpreted in the context of incomplete follow-up and potential follow-up-related selection bias. Fourth, although propensity matching was used to improve balance for the MyVal versus ACURATE neo2 comparison, residual confounding cannot be excluded because device selection in valve-in-valve TAVI is inherently anatomy dependent; therefore, the absence of statistically significant between-group differences should not be interpreted as evidence of equivalence or true comparability between platforms. Finally, the present study was not designed to assess structural valve durability beyond 1 year, and longer-term follow-up will be required to determine the persistence and clinical relevance of the observed hemodynamic findings.

## 5. Conclusions

In this prospective real-world cohort, transfemoral MyVal valve-in-valve TAVI was associated with favorable procedural safety and 1-year hemodynamic performance. Residual hemodynamic burden was driven primarily by surgical valve true internal diameter, supporting an anatomy-based valve-in-valve strategy with selective use of adjunctive optimization in constrained anatomies.

## Figures and Tables

**Table 1:** Baseline characteristics of the MyVal ViV-TAVI cohort.

Variable	Overall (*n* = 68)
Age, years	77 ± 7
Female sex	35/68 (51.5%)
Body mass index, kg/m^2^	27.8 ± 4.6
EuroSCORE II, %	7.3 ± 1.8
Hypertension	53/68 (77.9%)
Dyslipidemia	45/68 (66.2%)
Diabetes mellitus	21/68 (30.9%)
Coronary artery disease	39/68 (57.4%)
Prior PCI	18/68 (26.5%)
Prior CABG	11/68 (16.2%)
Prior myocardial infarction	9/68 (13.2%)
Prior stroke/TIA	6/68 (8.8%)
Atrial fibrillation	24/68 (35.3%)
COPD	14/68 (20.6%)
Chronic kidney disease (eGFR < 60 mL/min/1.73 m^2^)	27/68 (39.7%)
eGFR, mL/min/1.73 m^2^	61 ± 18
Prior permanent pacemaker	7/68 (10.3%)
NYHA class III	42/68 (62%)
NYHA class IV	26/68 (38%)
Time from SAVR to degeneration, years	10.6 ± 3.9
Failure mechanism: stenosis	34/68 (50.0%)
Failure mechanism: regurgitation	15/68 (22.1%)
Failure mechanism: mixed	19/68 (27.9%)
Surgical valve true internal diameter (ID), mm	22 ± 3
True ID ≤ 21 mm	24/68 (35.3%)
True ID > 21 mm	44/68 (64.7%)
Baseline mean gradient, mmHg	38.0 ± 9.5
Baseline peak gradient, mmHg	63.2 ± 15.1
Baseline AVA, cm^2^	0.80 ± 0.23
Baseline DVI	0.22 ± 0.06
Baseline EOAi, cm^2^/m^2^	0.45 ± 0.12
Baseline LVEF, %	53 ± 10

Footnote: Values are presented as mean ± SD or *n*/*N* (%). Abbreviations: AVA, aortic valve area; CABG, coronary artery bypass grafting; COPD, chronic obstructive pulmonary disease; DVI, Doppler velocity index; eGFR, estimated glomerular filtration rate; EOAi, effective orifice area index; ID, internal diameter; LVEF, left ventricular ejection fraction; NYHA, New York Heart Association; PCI, percutaneous coronary intervention; SAVR, surgical aortic valve replacement; SD, standard deviation; TIA, transient ischemic attack.

**Table 2:** Procedural characteristics and intraprocedural findings.

Variable	Overall (*n* = 68)
Access route: transfemoral	68/68 (100%)
General anesthesia	19/68 (27.9%)
Predilation	29/68 (42.6%)
Postdilation	18/68 (26.5%)
Surgical valve fracture	16/68 (23.5%)
Coronary protection used	8/68 (11.8%)
Chimney stenting performed	4/68 (5.9%)
Second valve implantation	0/68 (0.0%)
Emergency conversion to surgery	0/68 (0.0%)
Unresolved intraprocedural coronary flow compromise	0/68 (0.0%)
Procedure duration, min	74 ± 20
Fluoroscopy time, min	16.7 ± 6.5
Contrast volume, mL	108 ± 36

Footnote: Values are presented as mean ± SD or *n*/*N* (%). Abbreviations: mL, milliliters; min, minutes; SD, standard deviation.

**Table 3:** VARC-3 procedural success and 30-day outcomes.

Outcome	Value (*n* = 68)
Technical success (VARC-3)	68/68 (100.0%)
Early safety composite at 30 days (VARC-3-based composite)	4/68 (5.9%)
All-cause mortality (30 days)	0/68 (0.0%)
Stroke (all)	0/68 (0.0%)
Myocardial infarction	0/68 (0.0%)
Major vascular complication	1/68 (1.5%)
Major/life-threatening bleeding	1/68 (1.5%)
AKI stage 2–3	1/68 (1.5%)
New permanent pacemaker implantation	2/68 (2.9%)
Valve dysfunction requiring repeat procedure	0/68 (0.0%)
Valve-related reintervention (30 days)	0/68 (0.0%)

Footnote: Values are presented as *n*/*N* (%). Abbreviations: AKI, acute kidney injury; VARC-3, Valve Academic Research Consortium-3.

**Table 4:** Subgroup analysis by surgical valve true ID (≤21 mm vs. >21 mm).

Variable	True ID ≤ 21 mm (Overall n = 24)	True ID > 21 mm (Overall n = 44)	*p*-Value
Baseline mean gradient, mmHg	39.6 ± 9.8	37.1 ± 9.3	0.31
Post-procedure mean gradient, mmHg	7.2 ± 2.2	6.4 ± 2.0	0.14
30-day mean gradient, mmHg	8.2 ± 2.5	7.1 ± 2.2	0.08
1-year mean gradient, mmHg *	9.1 ± 2.6	7.6 ± 2.2	0.012
Mean gradient ≥ 10 mmHg at 1 year *	9/22 (40.9%)	5/37 (13.5%)	0.026
Surgical valve fracture performed	14/24 (58.3%)	2/44 (4.5%)	<0.001
Coronary protection used	4/24 (16.7%)	4/44 (9.1%)	0.44
Chimney stenting	2/24 (8.3%)	2/44 (4.5%)	0.61
30-day early safety composite	2/24 (8.3%)	2/44 (4.5%)	0.61
All-cause mortality during follow-up	1/24 (4.2%)	2/44 (4.5%)	1.00
HF hospitalization during follow-up	2/24 (8.3%)	1/44 (2.3%)	0.28
Composite of all-cause mortality or HF hospitalization during follow-up †	3/24 (12.5%)	3/44 (6.8%)	0.66

Footnote: Values are presented as mean ± SD or *n*/*N* (%). * One-year echocardiographic endpoints are reported using patients with available 1-year echocardiography in each subgroup. † Composite endpoint denotes all-cause mortality or heart failure hospitalization during follow-up. Abbreviations: HF, heart failure; ID, internal diameter; SD, standard deviation.

**Table 5:** Small-valve subgroup (true ID ≤ 21 mm): descriptive outcomes according to fracture strategy.

Variable	Fracture (Overall *n* = 14)	No Fracture (Overall *n* = 10)	*p*-Value
Post-procedure mean gradient, mmHg	6.1 ± 1.7	7.6 ± 2.0	0.041
30-day mean gradient, mmHg	7.2 ± 2.0	9.1 ± 2.6	0.056
1-year mean gradient, mmHg *	8.0 ± 2.1	10.4 ± 2.7	0.028
Mean gradient ≥ 10 mmHg at 1 year *	3/13 (23.1%)	6/9 (66.7%)	0.079
Coronary protection used	3/14 (21.4%)	1/10 (10.0%)	0.61
30-day early safety composite	1/14 (7.1%)	1/10 (10.0%)	1.00
All-cause mortality during follow-up	0/14 (0.0%)	1/10 (10.0%)	0.42
HF hospitalization during follow-up	1/14 (7.1%)	1/10 (10.0%)	1.00
Composite of all-cause mortality or HF hospitalization during follow-up †	1/14 (7.1%)	2/10 (20.0%)	0.55

Footnote: Values are presented as mean ± SD or *n*/*N* (%). * One-year echocardiographic endpoints are reported using patients with available 1-year echocardiography in each subgroup. † Composite endpoint denotes all-cause mortality or heart failure hospitalization during follow-up. Because fracture was selected non-randomly according to anatomical and procedural considerations, between-group differences should be interpreted as descriptive rather than causal. Abbreviations: HF, heart failure; ID, internal diameter; SD, standard deviation.

**Table 6:** Predictors of elevated residual gradient at 1 year (mean gradient ≥ 10 mmHg): Firth logistic regression (*n* = 59, events = 14).

Variable	Univariable OR (95% CI)	*p*-Value	Multivariable Adjusted OR (95% CI)	*p*-Value
True ID (per 1 mm decrease)	1.38 (1.10–1.79)	0.010	1.33 (1.04–1.78)	0.028
True ID ≤ 21 mm	3.80 (1.19–12.6)	0.025	—	—
Baseline mean gradient (per 5 mmHg increase)	1.12 (0.82–1.53)	0.46	1.09 (0.77–1.50)	0.61
Failure mechanism (stenosis vs. non-stenosis)	1.29 (0.42–4.08)	0.66	1.21 (0.37–4.01)	0.75
Surgical valve fracture (yes vs. no)	0.54 (0.16–1.73)	0.29	0.49 (0.12–1.71)	0.27
Postdilation	0.73 (0.21–2.36)	0.60	—	—
True ID × fracture interaction (exploratory)	—	—	*p* (interaction) = 0.12	—

Footnote: Values are reported as ORs with 95% CIs. The multivariable model included true ID, baseline mean transvalvular gradient, failure mechanism category, and surgical valve fracture strategy. True ID was parameterized per 1-mm decrease. Firth penalized logistic regression was used because of the limited number of events. The exploratory true ID × fracture interaction was evaluated in a secondary model and was not included in the primary adjusted model. Abbreviations: CI, confidence interval; ID, internal diameter; OR, odds ratio.

**Table 7:** Matched comparative outcomes: MyVal vs. ACURATE neo2 (38 matched pairs).

Endpoint	MyVal (*n* = 38)	ACURATE neo2 (*n* = 38)	Effect Estimate (95% CI)	*p*-Value
Technical success	38 (100.0%)	37 (97.4%)	OR not estimable (near-complete success)	0.31
30-day early safety composite	2 (5.3%)	3 (7.9%)	OR 0.65 (0.09–3.96)	0.64
30-day all-cause mortality	0 (0.0%)	0 (0.0%)	—	—
30-day stroke	0 (0.0%)	0 (0.0%)	—	—
30-day myocardial infarction	0 (0.0%)	0 (0.0%)	—	—
New permanent pacemaker implantation	1 (2.6%)	0 (0.0%)	OR 3.1 (0.12–170)	1.00
Major vascular complication	1 (2.6%)	0 (0.0%)	OR 3.1 (0.12–170)	1.00
Coronary protection used	6 (15.8%)	2 (5.3%)	OR 3.38 (0.62–23.0)	0.16
Chimney stenting	3 (7.9%)	2 (5.3%)	OR 1.54 (0.20–14.9)	0.65
Post-procedure mean gradient, mmHg	6.7 ± 2.0	6.9 ± 1.6	MD −0.2 (−1.0 to 0.6)	0.62
30-day mean gradient, mmHg	7.5 ± 2.2	7.8 ± 1.9	MD −0.3 (−1.2 to 0.6)	0.48
1-year mean gradient, mmHg	8.1 ± 2.4	8.0 ± 1.8	MD +0.1 (−0.9 to 1.1)	0.84
Mean gradient ≥ 10 mmHg at 1 year	8 (21.1%)	9 (23.7%)	OR 0.86 (0.28–2.63)	0.79
>mild PVL at 1 year	1 (2.6%)	1 (2.6%)	OR 1.00	1.00
1-year survival	94.7%	97.4%	Exploratory HR 1.86 (0.17–20.6)	0.62
HF hospitalization	2 (5.3%)	1 (2.6%)	Exploratory HR 2.03 (0.18–22.8)	0.56
Composite death/HF hospitalization	4 (10.5%)	2 (5.3%)	Exploratory HR 2.01 (0.37–10.8)	0.41
Post-ViV coronary access feasibility (attempted)	14/14 (100.0%)	10/11 (90.9%)	OR not stable (sparse)	0.26

Footnote: Values are presented as mean ± SD, *n*/*N* (%), or Kaplan–Meier estimates. Effect estimates are reported as matched ORs for binary fixed-time outcomes, paired MDs for continuous fixed-time outcomes, and exploratory HRs for time-to-event outcomes. Time-to-event analyses were performed using Cox proportional hazards models stratified by matched pair. One-year survival values represent Kaplan–Meier estimates. Abbreviations: CI, confidence interval; HF, heart failure; HR, hazard ratio; MD, mean difference; OR, odds ratio; PVL, paravalvular leak; SD, standard deviation; ViV, valve-in-valve.

## Data Availability

The data presented in this study are available on request from the corresponding author. The data are not publicly available due to ethical restrictions.

## Supplementary Materials

The following supporting information can be downloaded at: https://www.mdpi.com/article/10.3390/jcm15093462/s1, Table S1: Surgical valve characteristics of the MyVal ViV-TAVI cohort; Table S2: Distribution of preprocedural CT planning parameters and coronary-risk features; Table S3: Baseline covariate balance before and after propensity-score matching; Table S4: Sensitivity analyses for the exploratory MyVal versus ACURATE neo2 comparison; Table S5: Patient-level summary of adverse events during follow-up; Figure S1: Love plot of standardized mean differences before and after propensity-score matching; Figure S2: Propensity-score distributions and region of common support; Figure S3: Effect of surgical valve fracture on residual hemodynamic burden in small surgical valves (true ID ≤ 21 mm).

## Abbreviations

The following abbreviations are used in this manuscript:

AKI	Acute kidney injury
AVA	Aortic valve area
BVF	Bioprosthetic valve fracture
CABG	Coronary artery bypass grafting
CI	Confidence interval
COPD	Chronic obstructive pulmonary disease
CT	Computed tomography
DVI	Doppler velocity index
eGFR	Estimated glomerular filtration rate
EOAi	Effective orifice area index
HF	Heart failure
HR	Hazard ratio
ID	Internal diameter
LVEF	Left ventricular ejection fraction
MD	Mean difference
NYHA	New York Heart Association
OR	Odds ratio
PCI	Percutaneous coronary intervention
PPM	Prosthesis-patient mismatch
PVL	Paravalvular leak
SAVR	Surgical aortic valve replacement
SD	Standard deviation
TAVI	Transcatheter aortic valve implantation
TIA	Transient ischemic attack
VARC-3	Valve Academic Research Consortium-3
ViV	Valve-in-valve
ViV-TAVI	Valve-in-valve transcatheter aortic valve implantation
VIVID	Valve-in-Valve International Data
