# Supplementary material for: Transfemoral Valve-in-Valve TAVI with MyVal for Failed Surgical Aortic Bioprostheses: Procedural Outcomes, Serial Hemodynamics, and Anatomy-Based Determinants of Residual Gradient
Source: J Clin Med. 2026 May 1;15(9):3462. doi: 10.3390/jcm15093462 (PMC13163781; doi:10.3390/jcm15093462)
Supplement: Supplementary file 1 [file jcm-15-03462-s001.zip › jcm-4270457-supplementary.pdf]

Supplementary Methods: Propensity score methodology for exploratory internal device-platform comparison (MyVAL vs ACURATE neo2)

Rationale and analytic objective

An exploratory internal comparative analysis was performed to contextualize the performance of MyVAL in valve-in-valve (ViV) TAVI against an institutional cohort treated with ACURATE neo2. Because device choice was not randomized and was influenced by anatomical complexity, coronary obstruction risk, and treatment era, crude comparisons were considered susceptible to confounding by indication and temporal confounding. Propensity score methods were therefore used to improve exchangeability between device groups within the region of covariate overlap and to provide hypothesis-generating comparative estimates.

Study population for comparative analysis

The comparative dataset consisted of all transfemoral ViV TAVI procedures treated with MyVAL during the study period and a non-overlapping internal cohort of transfemoral ViV TAVI procedures treated with ACURATE neo2 in the same institution. For comparative analyses, the index procedure date was defined as the date of ViV implantation. Baseline covariates were defined using the pre-procedural assessment closest to the index procedure and prior to device selection. Patients with missing device identifier or missing key baseline covariates required for propensity estimation were handled as described below (Missing data and sensitivity analyses). No post-treatment variables (i.e., procedural or post-procedural variables potentially affected by device selection) were included in the propensity model.

Propensity score estimation

The propensity score was defined as the conditional probability of receiving MyVAL rather than ACURATE neo2 given observed baseline covariates. Propensity scores were estimated using multivariable logistic regression with device assignment (MyVAL vs ACURATE neo2) as the dependent variable. Covariates were selected a priori based on clinical plausibility and known determinants of ViV outcomes and device choice, emphasizing anatomical and era-related factors. The prespecified covariate set included:

Clinical variables: age, sex, body mass index, EuroSCORE II, estimated glomerular filtration rate (or chronic kidney disease category), left ventricular ejection fraction, atrial fibrillation, coronary artery disease, prior percutaneous coronary intervention, prior coronary artery bypass grafting, prior stroke/transient ischemic attack, chronic obstructive pulmonary disease, and baseline NYHA class.

Valve and hemodynamic variables: predominant failure mechanism (stenosis, regurgitation, mixed), baseline mean transvalvular gradient, baseline aortic valve area, and surgical valve true internal diameter (continuous).

Anatomical/coronary-risk variables: coronary obstruction-risk phenotype (predefined), including CT-derived measures and/or surgical prosthesis features associated with coronary compromise (e.g., low coronary height, restricted sinus/STJ dimensions, unfavorable valve-to-coronary geometry, and high-risk surgical valve design characteristics).

Treatment era: procedure date (or pre-specified era indicator) was incorporated to account for temporal changes in platform availability and institutional practice.

Continuous variables were retained as continuous whenever possible to avoid information loss. Where non-linearity was suspected and sample size permitted, restricted cubic splines (3–4 knots) were considered for key continuous predictors (notably surgical valve true ID and baseline gradient), with model parsimony maintained to avoid overfitting. Multicollinearity was assessed using variance inflation factors and clinical redundancy review; highly collinear covariates were managed by retaining the clinically dominant measure.

Model discrimination and calibration were assessed descriptively (c-statistic and calibration plots), not as a goal in itself but to ensure stable estimation of treatment probabilities. Predicted propensity scores were extracted on the probability scale for matching and diagnostics.

Common support and overlap assessment

Before matching, overlap between device groups was evaluated by visual inspection of propensity score distributions and by identifying the empirical region of common support (overlap in the observed propensity ranges). Patients with propensity scores outside the overlap region were considered non-comparable and were excluded from the matched analysis to reduce extrapolation beyond supported covariate space. Overlap diagnostics (distribution plots and balance summaries) are provided in the Supplementary Figures/Tables.

Matching algorithm

The primary comparative analysis used 1:1 nearest-neighbor propensity score matching without replacement. Matching was performed on the logit of the propensity score using a prespecified caliper width of 0.2 standard deviations of the logit propensity score, consistent with common methodological recommendations for reducing residual confounding while maintaining reasonable match yield. Greedy matching was used. The matched cohort size (number of pairs) was reported, and unmatched patients were retained for descriptive reporting but excluded from matched effect estimation.

Because a central concern was era-related confounding, sensitivity restriction analyses were pre-specified to evaluate robustness, including (i) matching within a restricted calendar period (when feasible) and/or (ii) including an explicit era indicator in the propensity model and assessing residual imbalance post-matching.

Balance diagnostics and reporting

Covariate balance was assessed using standardized mean differences (SMDs) computed before and after matching. Absolute SMD <0.10 was considered acceptable balance. Balance was reported using both tabular SMD summaries and Love plots for visual comparison of pre- versus post-match imbalance across covariates. In addition, variance ratios for key continuous covariates were inspected to identify residual distributional imbalance not captured by means alone. Where minor residual imbalance persisted in clinically important variables, sensitivity analyses using outcome models with covariate adjustment (doubly robust estimation) were performed as described below.

Comparative outcome analyses in the matched cohort

All comparative outcome analyses were explicitly labeled exploratory and hypothesis-generating. Analyses respected the matched-pair structure.

For binary outcomes, matched-pair methods were used: McNemar's test for paired proportions when applicable and exact matched tests (or conditional logistic regression) when sparse data limited asymptotic validity. Effect sizes were summarized as odds ratios with 95% confidence intervals when estimable; where event counts were too low for stable estimation, results were presented as paired event counts with exact p-values and confidence intervals where appropriate.

For continuous outcomes at single time points, paired t-tests were used for approximately normally distributed differences; otherwise, the Wilcoxon signed-rank test was used. Effect sizes were reported as paired mean differences with 95% confidence intervals (or median paired differences, as appropriate).

For serial haemodynamic outcomes (mean gradient and AVA), longitudinal mixed-effects models were fitted in the matched cohort with random intercepts for patient and fixed effects for time, device group, and the time×device interaction. The interaction term was used to assess whether haemodynamic trajectories differed between platforms over follow-up. Estimated marginal means were reported by time point and device group, and model-derived contrasts were used for time-specific comparisons when needed. Robust standard errors clustered at the patient level were used when appropriate to account for residual correlation and potential mild model misspecification.

Sensitivity analyses

Several sensitivity analyses were performed to examine robustness of the comparative findings to analytic choices and residual confounding.

First, overlap weighting was performed as a complementary propensity-based approach to preserve sample size while focusing inference on the region of maximal overlap between device groups. Overlap weights were constructed as 1−PS for treated (MyVAL) patients and PS for control (ACURATE neo2) patients. Weighted balance was assessed using weighted SMDs with the same <0.10 threshold. Comparative outcomes were then analyzed using weighted regression models appropriate to outcome type (weighted logistic for binary endpoints, weighted linear regression for continuous endpoints, and weighted mixed-effects models for serial outcomes).

Second, doubly robust estimation was used in which matched or weighted comparisons were supplemented by regression adjustment for any residual imbalanced covariates deemed clinically important (absolute post-adjustment SMD ≥0.10 or variables prioritized a priori).

Third, restricted-cohort sensitivity analyses were performed to reduce era-related confounding, including restricting analyses to time windows where both platforms were used contemporaneously or excluding early "selective-use" MyVAL cases when clinically justified.

All sensitivity analyses were interpreted qualitatively in relation to the primary matched results, emphasizing consistency of effect direction and magnitude rather than significance testing.

Missing data in propensity analyses

Baseline covariate missingness was evaluated before propensity estimation. There was no missingness in the key baseline covariates required for the final propensity model; accordingly, complete-case propensity estimation was used, and no imputation was necessary. As shown in Supplementary Table S2, no patients were excluded from the comparative dataset because of missing key propensity covariates.

Software

Propensity score estimation, matching, and diagnostics were performed in R using established packages for causal inference workflows (e.g., MatchIt for matching, cobalt for balance assessment, and, when applicable, WeightIt and survey for overlap weighting and weighted regression). Mixed-effects models were fitted using lme4/lmerTest and marginal means/contrasts obtained using emmeans. Exact or penalized methods for sparse binary outcomes were used when indicated (e.g., exact2x2, logistf/brglm2).

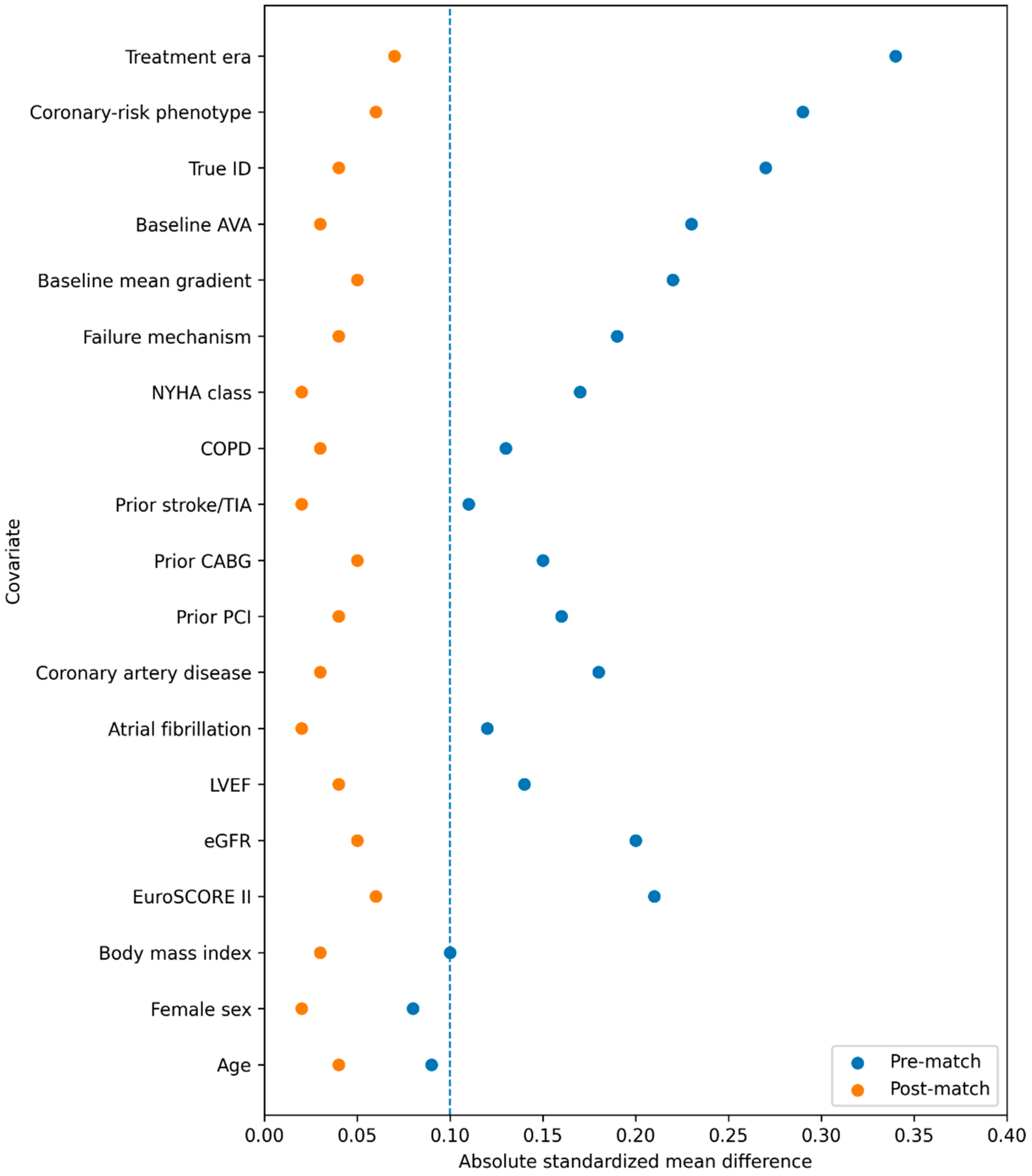

Supplementary Figure S1 Love plot of standardized mean differences before and after propensity-score matching

The figure displays absolute standardized mean differences for all prespecified propensity covariates before and after 1:1 nearest-neighbor matching. The dashed vertical line marks the prespecified threshold for acceptable measured balance (absolute standardized mean difference <0.10). All post-match covariates were below this threshold.

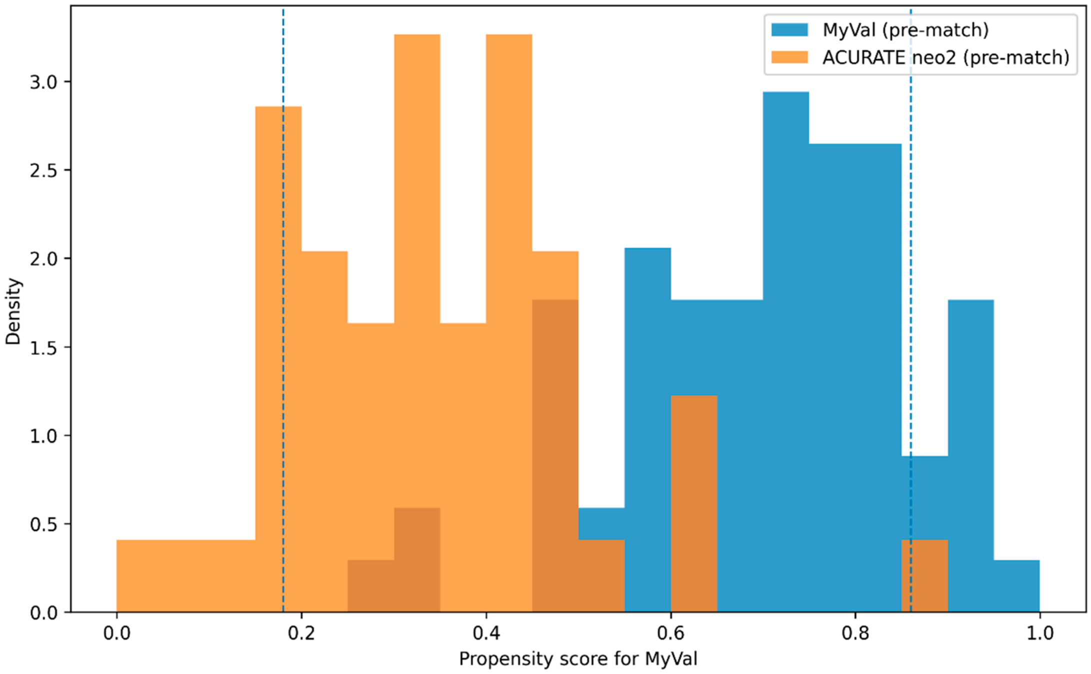

Supplementary Figure S2 Propensity-score distributions and region of common support

The figure shows pre-match propensity-score distributions for MyVal and ACURATE neo2, with the empirical region of common support indicated. Six MyVal cases and 3 ACURATE neo2 cases were excluded outside common support before matching.

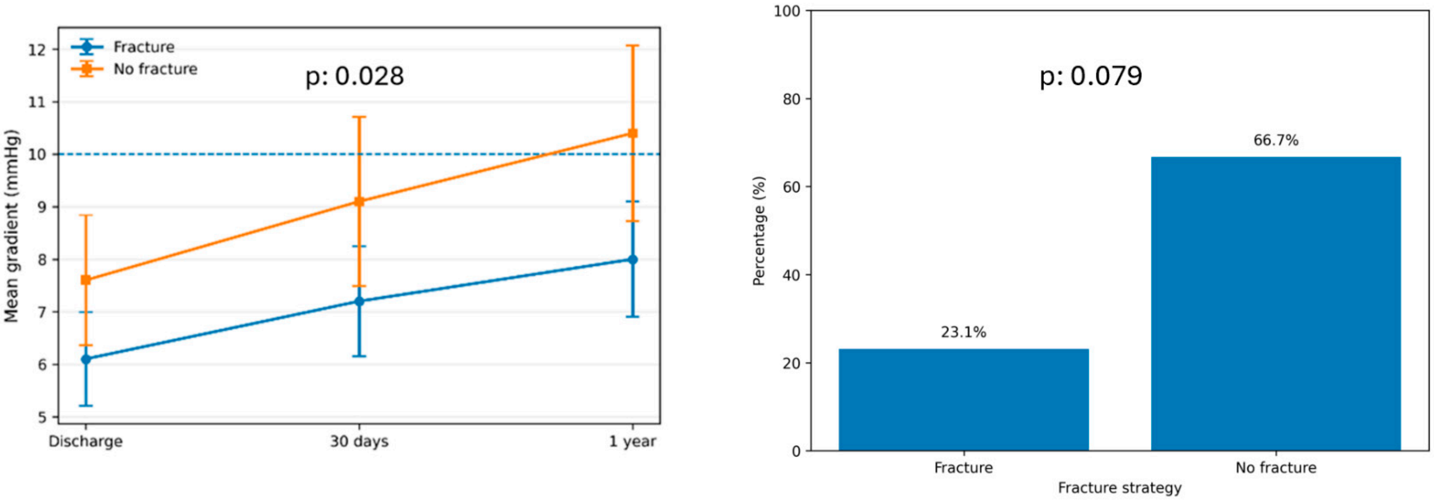

Supplementary Figure S3 Effect of surgical valve fracture on residual hemodynamic burden in small surgical valves (true ID ≤21 mm)

(A) Serial mean transvalvular gradient at discharge, 30 days, and 1 year in patients treated with and without surgical valve fracture after MyVal ViV-TAVI. Values are shown as mean ± SD. The dashed line indicates the 10 mmHg threshold. The p value refers to the between-group comparison of 1-year mean gradient. **(B)** Proportion of patients with 1-year mean transvalvular gradient ≥10 mmHg according to fracture strategy. The p value refers to the between-group comparison of the binary 1-year residual gradient endpoint.

Supplementary Table S1. Surgical valve characteristics of the MyVal ViV-TAVI cohort

Case	Surgical valve model	Labeled size (mm)	True ID (mm)	Valve architecture	Fluoroscopic marker characteristics	Leaflet design / mounting	Presumed fracture feasibility	Failure mode	Small-valve subgroup (true ID ≤21 mm)	Fracture strategy
1	Magna Ease	21	20	Stented	Radiopaque frame/posts visible	Internally mounted bovine pericardial leaflets	Likely fracturable	Stenosis	Yes	Fracture
2	Magna Ease	21	20	Stented	Radiopaque frame/posts visible	Internally mounted bovine pericardial leaflets	Likely fracturable	Mixed	Yes	Fracture
3	Perimount 2800	21	20	Stented	Radiopaque frame/posts visible	Internally mounted bovine pericardial leaflets	Likely fracturable	Stenosis	Yes	Fracture
4	Mosaic	21	19	Stented	Visible stent posts/frame	Stented porcine leaflet design	Likely fracturable	Stenosis	Yes	Fracture
5	Mitroflow	21	20	Stented	Limited fluoroscopic landmarks	Externally mounted pericardial leaflets	Likely fracturable	Mixed	Yes	Fracture
6	Biocor Epic	21	19	Stented	Visible stent posts/frame	Stented porcine leaflet design	Likely fracturable	Stenosis	Yes	Fracture
7	Trifecta	21	20	Stented	Limited fluoroscopic landmarks	Externally mounted pericardial leaflets / high leaflet profile	Not a routine BVF target	Regurgitation	Yes	Non-fracture
8	Mosaic	23	21	Stented	Visible stent posts/frame	Stented porcine leaflet design	Likely fracturable	Stenosis	Yes	Fracture
9	Biocor Epic	23	21	Stented	Visible stent posts/frame	Stented porcine	Likely fracturable	Stenosis	Yes	Fracture

Case	Surgical valve model	Labeled size (mm)	True ID (mm)	Valve architecture	Fluoroscopic marker characteristics	Leaflet design / mounting	Presumed fracture feasibility	Failure mode	Small-valve subgroup (true ID ≤21 mm)	Fracture strategy
						leaflet design				
10	Magna	21	20	Stented	Radiopaque frame/posts visible	Internally mounted bovine pericardial leaflets	Likely fracturable	Mixed	Yes	Fracture
11	Magna Ease	21	20	Stented	Radiopaque frame/posts visible	Internally mounted bovine pericardial leaflets	Likely fracturable	Stenosis	Yes	Fracture
12	Perimount 2800	21	20	Stented	Radiopaque frame/posts visible	Internally mounted bovine pericardial leaflets	Likely fracturable	Mixed	Yes	Fracture
13	Mitroflow	21	20	Stented	Limited fluoroscopic landmarks	Externally mounted pericardial leaflets	Likely fracturable	Stenosis	Yes	Fracture
14	Mosaic	23	21	Stented	Visible stent posts/frame	Stented porcine leaflet design	Likely fracturable	Regurgitation	Yes	Fracture
15	Biocor Epic	23	21	Stented	Visible stent posts/frame	Stented porcine leaflet design	Likely fracturable	Stenosis	Yes	Fracture
16	Trifecta	21	20	Stented	Limited fluoroscopic landmarks	Externally mounted pericardial leaflets / high leaflet profile	Not a routine BVF target	Regurgitation	Yes	Non-fracture

Case	Surgical valve model	Labeled size (mm)	True ID (mm)	Valve architecture	Fluoroscopic marker characteristics	Leaflet design / mounting	Presumed fracture feasibility	Failure mode	Small-valve subgroup (true ID ≤21 mm)	Fracture strategy
17	Magna	21	20	Stented	Radiopaque frame/posts visible	Internally mounted bovine pericardial leaflets	Likely fracturable	Stenosis	Yes	Non-fracture
18	Magna Ease	21	20	Stented	Radiopaque frame/posts visible	Internally mounted bovine pericardial leaflets	Likely fracturable	Mixed	Yes	Non-fracture
19	Perimount 2800	21	20	Stented	Radiopaque frame/posts visible	Internally mounted bovine pericardial leaflets	Likely fracturable	Stenosis	Yes	Non-fracture
20	Mosaic	23	21	Stented	Visible stent posts/frame	Stented porcine leaflet design	Likely fracturable	Stenosis	Yes	Non-fracture
21	Biocor Epic	23	21	Stented	Visible stent posts/frame	Stented porcine leaflet design	Likely fracturable	Mixed	Yes	Non-fracture
22	Mitroflow	21	20	Stented	Limited fluoroscopic landmarks	Externally mounted pericardial leaflets	Likely fracturable	Stenosis	Yes	Non-fracture
23	Trifecta	21	20	Stented	Limited fluoroscopic landmarks	Externally mounted pericardial leaflets / high leaflet profile	Not a routine BVF target	Regurgitation	Yes	Non-fracture

Case	Surgical valve model	Labeled size (mm)	True ID (mm)	Valve architecture	Fluoroscopic marker characteristics	Leaflet design / mounting	Presumed fracture feasibility	Failure mode	Small-valve subgroup (true ID ≤21 mm)	Fracture strategy
24	Magna Ease	21	20	Stented	Radiopaque frame/posts visible	Internally mounted bovine pericardial leaflets	Likely fracturable	Stenosis	Yes	Non-fracture
25	Magna Ease	23	22	Stented	Radiopaque frame/posts visible	Internally mounted bovine pericardial leaflets	Likely fracturable	Stenosis	No	Fracture
26	Magna Ease	23	22	Stented	Radiopaque frame/posts visible	Internally mounted bovine pericardial leaflets	Likely fracturable	Mixed	No	Non-fracture
27	Perimount 2800	23	22	Stented	Radiopaque frame/posts visible	Internally mounted bovine pericardial leaflets	Likely fracturable	Stenosis	No	Fracture
28	Mosaic	25	23	Stented	Visible stent posts/frame	Stented porcine leaflet design	Likely fracturable	Stenosis	No	Non-fracture
29	Mitroflow	23	22	Stented	Limited fluoroscopic landmarks	Externally mounted pericardial leaflets	Likely fracturable	Mixed	No	Non-fracture
30	Biocor Epic	25	23	Stented	Visible stent posts/frame	Stented porcine leaflet design	Likely fracturable	Stenosis	No	Non-fracture
31	Trifecta	23	22	Stented	Limited fluoroscopic landmarks	Externally mounted pericardial leaflets /	Not a routine BVF target	Regurgitation	No	Non-fracture

Case	Surgical valve model	Labeled size (mm)	True ID (mm)	Valve architecture	Fluoroscopic marker characteristics	Leaflet design / mounting	Presumed fracture feasibility	Failure mode	Small-valve subgroup (true ID ≤21 mm)	Fracture strategy
						high leaflet profile				
32	Magna	23	22	Stented	Radiopaque frame/posts visible	Internally mounted bovine pericardial leaflets	Likely fracturable	Stenosis	No	Non-fracture
33	Perimount 2800	25	24	Stented	Radiopaque frame/posts visible	Internally mounted bovine pericardial leaflets	Likely fracturable	Mixed	No	Non-fracture
34	Mosaic	27	25	Stented	Visible stent posts/frame	Stented porcine leaflet design	Likely fracturable	Stenosis	No	Non-fracture
35	Biocor Epic	27	25	Stented	Visible stent posts/frame	Stented porcine leaflet design	Likely fracturable	Regurgitation	No	Non-fracture
36	Mitroflow	25	24	Stented	Limited fluoroscopic landmarks	Externally mounted pericardial leaflets	Likely fracturable	Regurgitation	No	Non-fracture
37	Magna Ease	25	24	Stented	Radiopaque frame/posts visible	Internally mounted bovine pericardial leaflets	Likely fracturable	Stenosis	No	Non-fracture
38	Trifecta	25	24	Stented	Limited fluoroscopic landmarks	Externally mounted pericardial leaflets / high	Not a routine BVF target	Regurgitation	No	Non-fracture

Case	Surgical valve model	Labeled size (mm)	True ID (mm)	Valve architecture	Fluoroscopic marker characteristics	Leaflet design / mounting	Presumed fracture feasibility	Failure mode	Small-valve subgroup (true ID ≤21 mm)	Fracture strategy
						leaflet profile				
39	Magna	23	22	Stented	Radiopaque frame/posts visible	Internally mounted bovine pericardial leaflets	Likely fracturable	Mixed	No	Non-fracture
40	Perimount 2800	23	22	Stented	Radiopaque frame/posts visible	Internally mounted bovine pericardial leaflets	Likely fracturable	Stenosis	No	Non-fracture
41	Mosaic	25	23	Stented	Visible stent posts/frame	Stented porcine leaflet design	Likely fracturable	Stenosis	No	Non-fracture
42	Biocor Epic	25	23	Stented	Visible stent posts/frame	Stented porcine leaflet design	Likely fracturable	Mixed	No	Non-fracture
43	Mitroflow	23	22	Stented	Limited fluoroscopic landmarks	Externally mounted pericardial leaflets	Likely fracturable	Stenosis	No	Non-fracture
44	Trifecta	23	22	Stented	Limited fluoroscopic landmarks	Externally mounted pericardial leaflets / high leaflet profile	Not a routine BVF target	Regurgitation	No	Non-fracture
45	Magna Ease	23	22	Stented	Radiopaque frame/posts visible	Internally mounted bovine pericardial leaflets	Likely fracturable	Mixed	No	Non-fracture

Case	Surgical valve model	Labeled size (mm)	True ID (mm)	Valve architecture	Fluoroscopic marker characteristics	Leaflet design / mounting	Presumed fracture feasibility	Failure mode	Small-valve subgroup (true ID ≤21 mm)	Fracture strategy
46	Perimount 2800	25	24	Stented	Radiopaque frame/posts visible	Internally mounted bovine pericardial leaflets	Likely fracturable	Stenosis	No	Non-fracture
47	Mosaic	27	25	Stented	Visible stent posts/frame	Stented porcine leaflet design	Likely fracturable	Regurgitation	No	Non-fracture
48	Biocor Epic	27	25	Stented	Visible stent posts/frame	Stented porcine leaflet design	Likely fracturable	Stenosis	No	Non-fracture
49	Mitroflow	25	24	Stented	Limited fluoroscopic landmarks	Externally mounted pericardial leaflets	Likely fracturable	Mixed	No	Non-fracture
50	Magna	23	22	Stented	Radiopaque frame/posts visible	Internally mounted bovine pericardial leaflets	Likely fracturable	Stenosis	No	Non-fracture
51	Magna Ease	25	24	Stented	Radiopaque frame/posts visible	Internally mounted bovine pericardial leaflets	Likely fracturable	Stenosis	No	Non-fracture
52	Perimount 2800	23	22	Stented	Radiopaque frame/posts visible	Internally mounted bovine pericardial leaflets	Likely fracturable	Regurgitation	No	Non-fracture
53	Mosaic	25	23	Stented	Visible stent posts/frame	Stented porcine leaflet design	Likely fracturable	Mixed	No	Non-fracture

Case	Surgical valve model	Labeled size (mm)	True ID (mm)	Valve architecture	Fluoroscopic marker characteristics	Leaflet design / mounting	Presumed fracture feasibility	Failure mode	Small-valve subgroup (true ID ≤21 mm)	Fracture strategy
54	Biocor Epic	25	23	Stented	Visible stent posts/frame	Stented porcine leaflet design	Likely fracturable	Stenosis	No	Non-fracture
55	Mitroflow	23	22	Stented	Limited fluoroscopic landmarks	Externally mounted pericardial leaflets	Likely fracturable	Mixed	No	Non-fracture
56	Trifecta	25	24	Stented	Limited fluoroscopic landmarks	Externally mounted pericardial leaflets / high leaflet profile	Not a routine BVF target	Regurgitation	No	Non-fracture
57	Magna Ease	23	22	Stented	Radiopaque frame/posts visible	Internally mounted bovine pericardial leaflets	Likely fracturable	Stenosis	No	Non-fracture
58	Perimount 2800	25	24	Stented	Radiopaque frame/posts visible	Internally mounted bovine pericardial leaflets	Likely fracturable	Mixed	No	Non-fracture
59	Mosaic	27	25	Stented	Visible stent posts/frame	Stented porcine leaflet design	Likely fracturable	Stenosis	No	Non-fracture
60	Biocor Epic	27	25	Stented	Visible stent posts/frame	Stented porcine leaflet design	Likely fracturable	Regurgitation	No	Non-fracture
61	Mitroflow	25	24	Stented	Limited fluoroscopic landmarks	Externally mounted	Likely fracturable	Stenosis	No	Non-fracture

Case	Surgical valve model	Labeled size (mm)	True ID (mm)	Valve architecture	Fluoroscopic marker characteristics	Leaflet design / mounting	Presumed fracture feasibility	Failure mode	Small-valve subgroup (true ID ≤21 mm)	Fracture strategy
						pericardial leaflets				
62	Magna	25	24	Stented	Radiopaque frame/posts visible	Internally mounted bovine pericardial leaflets	Likely fracturable	Mixed	No	Non-fracture
63	Magna Ease	25	24	Stented	Radiopaque frame/posts visible	Internally mounted bovine pericardial leaflets	Likely fracturable	Stenosis	No	Non-fracture
64	Perimount 2800	23	22	Stented	Radiopaque frame/posts visible	Internally mounted bovine pericardial leaflets	Likely fracturable	Regurgitation	No	Non-fracture
65	Mosaic	25	23	Stented	Visible stent posts/frame	Stented porcine leaflet design	Likely fracturable	Mixed	No	Non-fracture
66	Biocor Epic	25	23	Stented	Visible stent posts/frame	Stented porcine leaflet design	Likely fracturable	Stenosis	No	Non-fracture
67	Mitroflow	23	22	Stented	Limited fluoroscopic landmarks	Externally mounted pericardial leaflets	Likely fracturable	Regurgitation	No	Non-fracture
68	Trifecta	23	22	Stented	Limited fluoroscopic landmarks	Externally mounted pericardial leaflets / high leaflet profile	Not a routine BVF target	Mixed	No	Non-fracture

Supplementary Table S2. Distribution of preprocedural CT planning parameters and coronary-risk features

**Variable**	**Overall (n=68)**	**Small-valve subgroup, true ID ≤21 mm (n=24)**	**True ID >21 mm (n=44)**
Left main coronary height, mm	11.1 ± 2.6	10.6 ± 2.4	11.4 ± 2.7
Right coronary height, mm	13.0 ± 2.8	12.5 ± 2.6	13.3 ± 2.9
Minimum coronary height, mm	10.4 ± 2.3	9.9 ± 2.1	10.7 ± 2.4
Sinus of Valsalva diameter, mm	30.8 ± 4.2	29.7 ± 3.9	31.4 ± 4.3
Sinotubular junction diameter, mm	27.5 ± 3.8	26.6 ± 3.5	28.0 ± 3.9
Minimum valve-to-coronary distance, mm	4.2 ± 1.7	3.8 ± 1.5	4.4 ± 1.8
Valve-to-sinotubular junction distance, mm	3.9 ± 1.6	3.5 ± 1.4	4.1 ± 1.7
Low coronary height (<10 mm), n/N (%)	18/68 (26.5%)	9/24 (37.5%)	9/44 (20.5%)
Restricted sinus/STJ anatomy, n/N (%)	14/68 (20.6%)	7/24 (29.2%)	7/44 (15.9%)
Unfavorable valve-to-coronary geometry, n/N (%)	16/68 (23.5%)	8/24 (33.3%)	8/44 (18.2%)
High coronary-risk phenotype, n/N (%)	12/68 (17.6%)	7/24 (29.2%)	5/44 (11.4%)
Coronary protection performed, n/N (%)	8/68 (11.8%)	4/24 (16.7%)	4/44 (9.1%)
Chimney stenting performed, n/N (%)	4/68 (5.9%)	2/24 (8.3%)	2/44 (4.5%)

Supplementary Table S3. Baseline covariate balance before and after propensity-score matching.

**Covariate**	**Pre-match absolute SMD**	**Post-match absolute SMD**	**Overlap-weighted absolute SMD**
Age	0.09	0.04	0.03
Female sex	0.08	0.02	0.02
Body mass index	0.10	0.03	0.03
EuroSCORE II	0.21	0.06	0.05
eGFR / CKD category	0.20	0.05	0.04
LVEF	0.14	0.04	0.03
Atrial fibrillation	0.12	0.02	0.02
Coronary artery disease	0.18	0.03	0.03
Prior PCI	0.16	0.04	0.03
Prior CABG	0.15	0.05	0.04
Prior stroke/TIA	0.11	0.02	0.02
COPD	0.13	0.03	0.03
Baseline NYHA class	0.17	0.02	0.02
Failure mechanism	0.19	0.04	0.03
Baseline mean gradient	0.22	0.05	0.04
Baseline AVA	0.23	0.03	0.03
Surgical valve true ID	0.27	0.04	0.03
Coronary-risk phenotype	0.29	0.06	0.05
Treatment era	0.34	0.07	0.06

Supplementary Table S4. Sensitivity analyses for the exploratory MyVal versus ACURATE neo2 comparison.

**Outcome**	**Primary 1:1 matched analysis**	**Overlap-weighted analysis**	**Doubly robust adjusted analysis**	**Restricted-era sensitivity analysis**	**Overall interpretation**
Post-procedure mean gradient	MD −0.2 (95% CI −1.0 to 0.6), p=0.62	MD −0.1 (95% CI −0.8 to 0.5), p=0.68	MD −0.2 (95% CI −0.9 to 0.5), p=0.59	MD −0.3 (95% CI −1.2 to 0.7), p=0.56	Concordant; no material difference
30-day mean gradient	MD −0.3 (95% CI −1.2 to 0.6), p=0.48	MD −0.2 (95% CI −1.0 to 0.5), p=0.54	MD −0.3 (95% CI −1.1 to 0.5), p=0.47	MD −0.4 (95% CI −1.4 to 0.7), p=0.43	Concordant; no material difference
1-year mean gradient	MD +0.1 (95% CI −0.9 to 1.1), p=0.84	MD +0.2 (95% CI −0.7 to 1.0), p=0.71	MD +0.1 (95% CI −0.8 to 1.0), p=0.81	MD +0.2 (95% CI −1.0 to 1.4), p=0.76	Concordant; no material difference
Mean gradient ≥10 mmHg at 1 year	OR 0.86 (95% CI 0.28–2.63), p=0.79	OR 0.91 (95% CI 0.34–2.31), p=0.84	OR 0.88 (95% CI 0.30–2.54), p=0.81	OR 0.95 (95% CI 0.26–3.18), p=0.92	Concordant; imprecise
Longitudinal mean-gradient trajectory (time×device interaction)	p=0.74	p=0.69	p=0.71	p=0.78	No evidence of differential trajectory
Composite death/HF hospitalization	HR 2.01 (95% CI 0.37–10.8), p=0.41	HR 1.82 (95% CI 0.39–8.40), p=0.45	HR 1.90 (95% CI 0.35–9.70), p=0.43	HR 1.74 (95% CI 0.29–9.05), p=0.52	Directionally similar; highly imprecise

Supplementary Table S5. Patient-level summary of adverse events during follow-up

Patient	Subgroup	Event type	Timing from ViV-TAVI	Valve status at/near event	Relation to valve status	Management / outcome
P1	Small-valve subgroup (true ID ≤21 mm)	All-cause mortality	2.3 months	Mild residual gradient; no >mild PVL; no evidence of thrombosis or endocarditis	Not clearly valve-related	Died during non-cardiac hospitalization
P2	Small-valve subgroup (true ID ≤21 mm)	HF hospitalization	4.8 months	Residual gradient elevated; no severe regurgitation; no valve thrombosis	Possibly multifactorial; contribution of residual hemodynamic burden cannot be excluded	Treated medically; discharged
P3	Small-valve subgroup (true ID ≤21 mm)	HF hospitalization	9.1 months	Mild-moderate residual gradient; no >mild PVL; no structural valve complication identified	Multifactorial; not definitive for valve failure	Diuretic intensification; no reintervention
P4	True ID >21 mm subgroup	All-cause mortality	3.7 months	Valve hemodynamics preserved on latest available echocardiography; no thrombosis or endocarditis	Not clearly valve-related	Sudden death of uncertain mechanism
P5	True ID >21 mm subgroup	All-cause mortality	10.4 months	Stable valve function on prior follow-up imaging; no valve-related complication documented	Not clearly valve-related	Died after non-cardiac clinical deterioration

Patient	Subgroup	Event type	Timing from ViV-TAVI	Valve status at/near event	Relation to valve status	Management / outcome
P6	True ID >21 mm subgroup	HF hospitalization	7.6 months	Mild residual gradient; no severe regurgitation; no evidence of thrombosis or endocarditis	Multifactorial; not clearly valve failure	Medical therapy adjustment; discharged
